# Therapeutic blood-brain barrier modulation and stroke treatment by a bioengineered FZD_4_-selective WNT surrogate in mice

**DOI:** 10.1038/s41467-023-37689-1

**Published:** 2023-06-02

**Authors:** Jie Ding, Sung-Jin Lee, Lukas Vlahos, Kanako Yuki, Cara C. Rada, Vincent van Unen, Meghah Vuppalapaty, Hui Chen, Asmiti Sura, Aaron K. McCormick, Madeline Tomaske, Samira Alwahabi, Huy Nguyen, William Nowatzke, Lily Kim, Lisa Kelly, Douglas Vollrath, Andrea Califano, Wen-Chen Yeh, Yang Li, Calvin J. Kuo

**Affiliations:** 1 Department of Medicine, Division of Hematology, Stanford University School of Medicine, Stanford, CA 94305 USA; 2 Surrozen, Inc. South San Francisco, South San Francisco, CA 94080 USA; 3 Department of Systems Biology, Columbia University, Columbia, NY 10032 USA; 4 Institute for Immunity, Transplantation and Infection, Stanford University School of Medicine, Stanford, CA 94305 USA; 5 Department of Genetics, Stanford University School of Medicine, Stanford, CA 94305 USA

**Keywords:** Target validation, Stroke, Stroke

## Abstract

Derangements of the blood-brain barrier (BBB) or blood-retinal barrier (BRB) occur in disorders ranging from stroke, cancer, diabetic retinopathy, and Alzheimer’s disease. The Norrin/FZD_4_/TSPAN12 pathway activates WNT/β-catenin signaling, which is essential for BBB and BRB function. However, systemic pharmacologic FZD_4_ stimulation is hindered by obligate palmitoylation and insolubility of native WNTs and suboptimal properties of the FZD_4_-selective ligand Norrin. Here, we develop L6-F4-2, a non-lipidated, FZD_4_-specific surrogate which significantly improves subpicomolar affinity versus native Norrin. In Norrin knockout (*Ndp*^*KO*^) mice, L6-F4-2 not only potently reverses neonatal retinal angiogenesis deficits, but also restores BRB and BBB function. In adult C57Bl/6J mice, post-stroke systemic delivery of L6-F4-2 strongly reduces BBB permeability, infarction, and edema, while improving neurologic score and capillary pericyte coverage. Our findings reveal systemic efficacy of a bioengineered FZD_4_-selective WNT surrogate during ischemic BBB dysfunction, with potential applicability to adult CNS disorders characterized by an aberrant blood-brain barrier.

## Abstract (web summary)

The WNT/b-catenin pathway is essential for bloodbrain barrier (BBB) and blood-retina barrier (BRB) function. A bioengineered FZD4-selective WNT surrogate demonstrated systemic efficacy during BRB and ischemic stroke BBB dysfunction in mice.

## Introduction

The cerebrovasculature comprises a highly specialized vascular bed that tightly regulates the movement of nutrients, ions, and cells from the blood into the brain parenchyma. This stringent cerebrovascular integrity is controlled by the blood-brain barrier (BBB) and blood-retinal barrier (BRB) to meet high metabolic demands, while specifically inhibiting transmigration of toxins and pathogens to prevent neuronal injury^1^. The BBB and BRB are comprised of a neurovascular unit (NVU) consisting of an endothelial cell (EC) layer with abundant tight junctions and covered by a dense pericyte layer, all engulfed by astrocyte end-feet^2^. BBB disruption and/or dysregulation is particularly significant to the pathology of numerous central nervous system (CNS) diseases including stroke, cancer, epilepsy, multiple sclerosis, ALS, Alzheimer’s disease, and recently, SARS-CoV-2 “brain fog”^3–8^.

Significant recent evidence has demonstrated essential WNT/β-catenin signaling regulation of both BBB/BRB function and CNS angiogenesis. Many ligands of the WNT family functionally crosslink Frizzled (FZD) 7-pass transmembrane/GPCR-like receptors to LRP5/6 co-receptors to initiate β-catenin signaling. Subsequently, inhibition of Axin-dependent degradation enables β-catenin nuclear translocation, association with LEF/TCF transcription factors and downstream target gene transactivation^9^. Within CNS endothelium, multiple inputs converge onto β-catenin signaling, as in the Norrin/FZD_4_/TSPAN12 and WNT7/GPR124/RECK pathways^10^. Mouse and zebrafish mutations in the aforementioned signaling nodes inhibit endothelial WNT/β-catenin signaling, with overlapping embryonic lethal phenotypes of impaired brain angiogenesis, glomeruloid vascular malformations, hemorrhage and BBB/BRB immaturity and leakage^11–14^. Importantly, compromise of endothelial or generalized WNT/β-catenin signaling exacerbates disease pathology in adult mice with hemorrhagic transformation of experimental stroke and glioma, with reversal by genetic WNT/β-catenin signaling activation^15–17^.

Frizzled-4 (FZD_4_) is particularly relevant for BBB/BRB regulation. FZD_4_ is highly expressed in CNS endothelium, while deletion in retinal or cerebellar EC elicits BRB compromise and retinal angiogenesis deficits in neonates, and cerebellar BBB leakage in juvenile mice^10,11,18^. The secreted protein Norrin, encoded by *Ndp*, exhibits remarkable binding specificity for FZD_4_, but not other FZD family members. Despite lack of homology to classical WNTs, Norrin potently triggers FZD_4_ ubiquitination and internalization^19^ to activate β-catenin-dependent WNT signaling through co-receptors LRP5/6 and TSPAN12^20,21^, while ectopic transgenic Norrin production restores cerebellar BBB integrity in *Ndp*^*KO*^ mice^18^. Mutation of *Ndp*^22,23^, *Fzd4*^21,24^, *Lrp5*^25,26^, or *Tspan12*^27,28^ compromises EC WNT/β-catenin signaling, with neonatal ocular manifestations of impaired retinal angiogenesis, persistent hyaloid vessels, BRB leakage, and cerebellar BBB permeability. These mutations also underlie human vitreoretinal diseases, including Norrie disease, familial exudative vitreoretinopathy, and Coats’ disease^29,30^.

Despite significant potential, successful pharmacologic FZD_4_ activation for therapeutic BBB modulation has remained elusive. Norrin is a FZD_4_-selective surrogate but is a short-range signal that is poorly secreted and highly associated with the extracellular matrix^31,32^. Unlike WNTs, which robustly activate WNT/β-catenin signaling with FZDs in the absence of co-transfected *LRPs*, Norrin requires both FZD_4_ and LRP5/6, implying that Norrin interacts in a ternary complex with FZD_4_ and LRPs^31,32^. WNT proteins themselves are obligately palmitoylated, which restricts expression, solubility, and in vivo pharmacokinetics^33,34^. In contrast, we previously developed bioengineered lipid-free FZD-specific WNT surrogates that signal by crosslinking FZD and LRP5/6 via anti-FZD single-chain antibodies (scFv) fused to LRP5/6-binding scFv or DKK1 C-terminal domains^35,36^. We further designed an array of FZD-selective surrogates active in a narrow dosing window, including monoselective FZD_4_ ligands that regulated hepatic zonation and intestinal growth in vivo^37,38^. FZD_4_-selective WNT surrogates also regulate BRB function in neonatal *Tspan12*^*−/−*^ mice with partial rescue at 2–3 months of age^28^, and in postnatal oxygen-induced retinopathy models in WT mice^39^.

Here, we describe an improved FZD_4_-selective WNT surrogate (L6-F4-2) with subpicomolar affinity representing multi-log affinity improvements over Norrin and previously reported FZD_4_ surrogates^28,37^. L6-F4-2 bioactivity was confirmed in cultured brain EC and by in vivo rescue of neonatal retinal BRB function and angiogenesis defects in *Ndp*^*KO*^ mice. L6-F4-2 also successfully treated blood-brain barrier pathology in mature mice by rescue of *Ndp*^*KO*^ cerebellar BBB leakage. We extended these findings to adult blood-brain barrier function using ischemic stroke as a model of prevalent disease, where post-stroke administration of L6-F4-2 reversed BBB permeability, infarction, edema, and neurologic score. Overall, these studies develop a highly optimized FZD_4_-selective WNT surrogate and suggest that FZD_4_ stimulation could be of potential use in treating pathologic BBB compromise in neurological diseases.

## Results

### Generation of a subpicomolar affinity monospecific FZD_4_-selective WNT surrogate, L6-F4-2

**Fig. 1:**
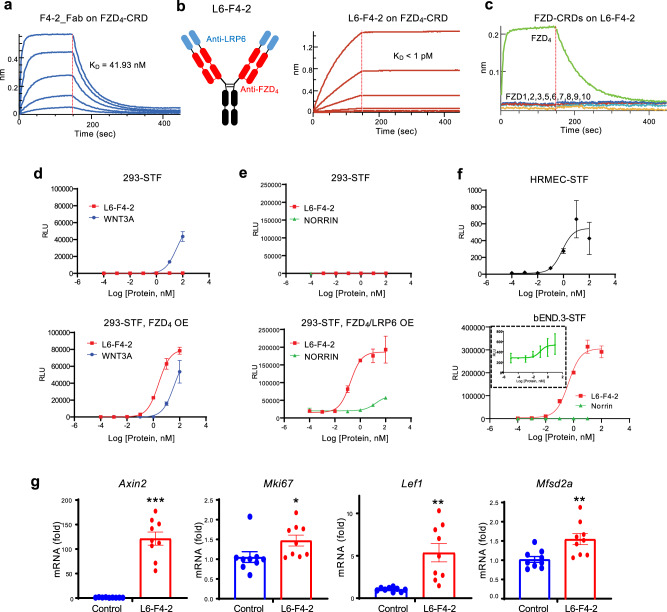
Characterization of the monoFZD_4_-specific WNT/β-catenin signaling surrogate, L6-F4-2. **a** Binding affinity of the recombinant F4-2_Fab molecule to FZD_4_ CRD measured by BLI assay. Dotted lines indicate the global fits generated by using a 1:1 Langmuir binding model. **b** Schematic of L6-F4-2 and the binding affinity of the L6-F4-2 molecule to the FZD_4_ CRD measured by BLI assay. Dotted lines indicate the global fits generated by using a 1:1 Langmuir binding model. **c** The binding specificity of L6-F4-2 against all ten FZD CRDs was examined by BLI assay. **d** Dose-dependent STF activity of L6-F4-2 or WNT3A in 293STF cells (top) or FZD_4_-transfected 293STF cells (bottom, OE = overexpression). **e** Dose-dependent STF activity of L6-F4-2 or Norrin in 293STF cells (top) or cells transfected with both FZD_4_ and LRP6 (bottom, OE = overexpression). **f** Dose-dependent STF activities of L6-F4-2 in HRMEC cells (top) and bEnd.3 cells (bottom). Inset box in the bEnd.3-STF graph is an enlarged plot of the Norrin response. **g** Quantitative RT-PCR of *Axin2*, *Mki67*, *Lef1* and *Mfsd2a* gene expression in bEnd.3 cells. mRNA expression values were normalized by *Actb* gene expression. Results are from three independent experiments. Graphs are shown as mean ± SEM; *p < 0.05; **p < 0.01; ***p < 0.001, two- sided Mann–Whitney *U* test. Source data are provided as a Source Data file.

Given the genetic evidence supporting the importance of FZD_4_ in retinal vascular biology and BBB/BRB function, we generated a FZD_4_/LRP6-specific WNT mimetic molecule to crosslink these targets and initiate WNT/β-catenin signaling. The FZD_4_ binder selected was 5063, hereafter F4-2 (WO 2019/159084 A1). The fragment antigen-binding (Fab) form of F4-2 binding to the cysteine-rich domain (CRD) of FZD_4_ was confirmed by biolayer interferometry. The dissociation constant (K_D_) of the monovalent binding was observed as ~42 nM (Fig. 1a). As we and others previously showed that tetravalent bi-specific antibodies with two FZD and two LRP binding arms are highly potent and efficacious WNT mimetics in inducing β-catenin signaling^40,41^, we assembled F4-2 with the LRP6 binder, YW211.31.57^41^ (hereafter L6), in the tetravalent antibody format. This is referred to as L6-F4-2, where L6 is fused to the N-terminus of F4-2 IgG (Fig. 1b). Due to the avidity effect from the bivalent binding, L6-F4-2 showed high affinity binding toward FZD_4_-CRD with *K*_D_ < 1 pM (Fig. 1b), which strongly surpasses previously described affinity measurements of native Norrin or previously described FZD_4_ surrogates (11–260 nM)^42,43^. L6-F4-2 FZD_4_ specificity was also preserved in the tetravalent format (Fig. 1c).

The ability of L6-F4-2 to activate WNT/β-catenin signaling was assessed in WNT-responsive HEK293 Super TOP-FLASH (STF) reporter cells^36^ (293STF assay). While L6-F4-2 did not induce a strong response in parental 293STF cells due to low levels of endogenous FZD_4_, L6-F4-2 strongly and dose-dependently activated WNT/β-catenin signaling in FZD_4_-transfected 293STF cells (Fig. 1d). This activity was stronger than recombinant WNT3A (Fig. 1d) and recombinant Norrin in 293STF cells with FZD_4_ and LRP6 overexpression (Fig. 1e). A distinct monospecific FZD_4_-selective WNT surrogate, F4L5.13, was reported using an alternative molecular format, with dumbbell-like structures against two FZD_4_ and one or two LRP5 molecules and exhibited comparable maximal STF response to recombinant Norrin^28^. However, our monospecific FZD_4_ activator, L6-F4-2, was ~100 times more potent (EC_50_, L6-F4-2 0.1658 nM vs. Norrin 16.35 nM) with ~30 times higher E_max_ than Norrin (Fig. 1e). Though these different WNT mimetics were not directly compared side-by-side nor against the same Norrin preparation, these results suggest that affinity, appropriate molecular format, and geometry are critical for WNT mimetic generation^41^. We further performed STF assays in human retinal microvascular endothelial cells (HRMEC) and murine brain microvascular endothelial cells (bEND.3). As both of these EC lines express FZD_4_, L6-F4-2-induced dose-dependent STF responses in each without requiring exogenous FZD_4_ transfection (Fig. 1f). To further confirm the induction of WNT/β-catenin signaling, expression of the WNT target genes *Axin2, Lef1* and *Mfsd2a*^44–46^ as well as *Mki67* were assessed. L6-F4-2 significantly enhanced these mRNAs in bEND.3 cells (Fig. 1g) as well as *Apcdd1* and *Cldn5* in cultured primary mouse brain endothelium (Supplementary Fig. 1).

### L6-F4-2 treatment rescues retinopathy in *Ndp*^*KO*^ mice

**Fig. 2:**
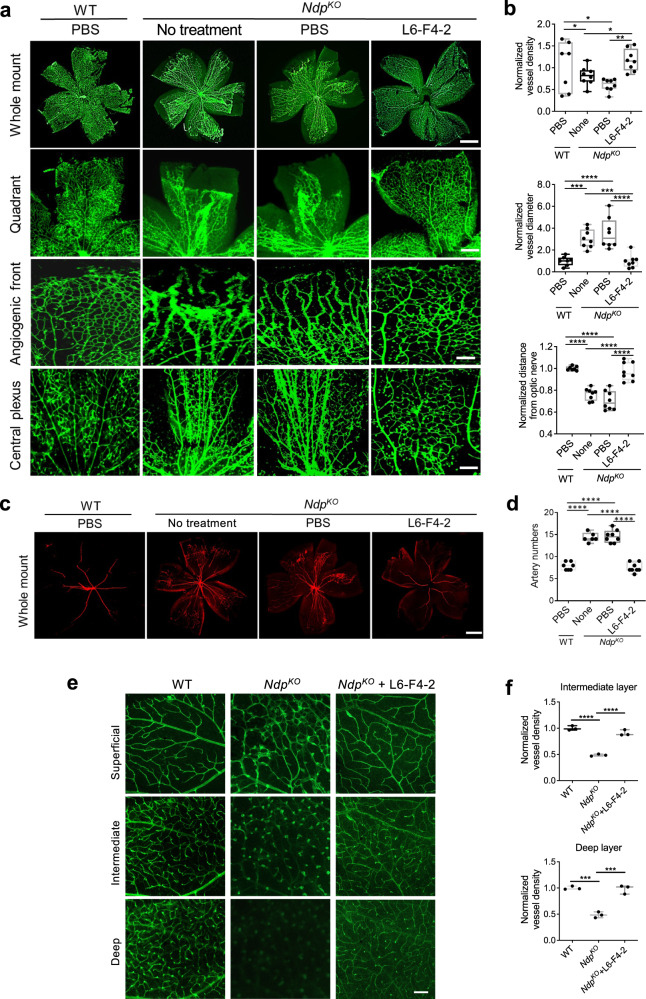
L6-F4-2 treatment rescues retinopathy in *Ndp*^*KO*^ mice. **a**–**d** WT (control) and *Ndp*^*KO*^ male and female mice were treated with PBS or L6-F4-2 by single intravitreal injection (0.19 μg) at P0 and retinas were harvested at P8. **a** Whole-mount isolectin GS-IB4 labeling of retinal veins. Representative images of the whole retinal vascular plexus, quadrant vascular plexus, angiogenic front and central plexus are shown. Scale bars represent 0.5 mm (top), 0.3 mm (middle) and 0.15 mm (bottom two rows). **b** Quantification of vessel diameter, vessel density and distance from the optic nerve from (**a**). **c** Whole-mount anti-smooth muscle actin labeling of retinal arteries at P8. **d** Quantification of artery branches from (**c**). Scale bar represents 0.5 mm. In all the quantifications, error bars represent mean ± SEM, *Ndp* WT *n* = 7 mice, *Ndp*^*KO*^ *n* = 8 mice, *p < 0.05, **p < 0.01, ***p < 0.001; two-sided Mann–Whitney *U* test. **e** WT (control) and *Ndp*^*KO*^ mice were treated with PBS or L6-F4-2 by intravitreal injection (0.19 μg) at P5 and every three days with retina harvest at P21. Optical sections of isolectin-stained P21 retinas showing the three-layered retinal vasculature (superficial, intermediate, and deep layers) to document vascular architecture. Scale bars represent 100 μm. **f** Vascular density quantification of retina intermediate and deep layers from (**e**), *n* = 3 mice, ***p < 0.001, ****p < 0.0001, one-way ANOVA. Source data are provided as a Source Data file. For box plots, whiskers indicate the minimum and maximum values in the dataset. The center is the median number. Boundaries of boxes are the first quartile and third quartile.

Norrin uniquely binds the FZD_4_ receptor, which is essential for neonatal retinal vascular development, to activate WNT/β-catenin signaling^21^. In humans, mutation of *Ndp*, encoding Norrin, causes Norrie disease with congenital blindness^47^. *Ndp*^*KO*^ mice exhibit severely impaired neonatal retinal angiogenesis with lack of capillary networks^23^, defective EC proliferation and abnormal artery-vein crossings^18^. Similarly, disruption of Norrin/FZD_4_ signaling by conditional FZD_4_ knockout in ECs reduces neonatal retinal vascularization^24^. We thus tested if our L6-F4-2 FZD_4_-selective WNT surrogate could substitute for the absence of Norrin in *Ndp*^*KO*^ mice and rescue the mutant retinal vasculature phenotype. We administered L6-F4-2 or PBS by single intravitreal (IVT) injection into the left eye at postnatal day 0 (P0) and used the right eye as a non-treatment control. P8 retinas were harvested, and veins were stained with isolectin GS-IB4, revealing that PBS-injected or non-injected mice displayed characteristic *Ndp*^*KO*^ phenotypes with reduced vein distance from the optic nerve, enlarged vein diameter and loss of fine capillary networks. These impaired vein vasculatures were completely rescued by L6-F4-2 treatment of *Ndp*^*KO*^ mice, which then resembled *Ndp* WT retinal veins (Fig. 2a, b).

In contrast to severely atretic retinal vein development, *Ndp*^*KO*^ mice also manifest increased neonatal retinal arteries^18^. Whole-mount arterial staining of neonatal retinas with anti-smooth muscle actin revealed that single IVT injection of L6-F4-2 restored the increased arterial phenotype of *Ndp*^*KO*^ mice to *Ndp* WT levels (Fig. 2c, d). Optical sections were used to document malformation of the three-layered retinal vasculature. Control mice injected with PBS displayed characteristic phenotypes of *Ndp*^*KO*^ mice including absence of intraretinal capillaries in the deep layer and glomeruloid vascular malformations instead of a properly formed capillaries in the intermediate layer. Angiogenesis was restored and intermediate and deep intraretinal capillary beds were properly formed in *Ndp*^*KO*^ mice injected with L6-F4-2 (Fig. 2e). The vascular density of retina intermediate and deep layers was quantified in Fig. 2f.

In WT retinas, major arteries and veins are segregated into alternating and radially arrayed territories, and rarely cross. By contrast, P8 *Ndp*^*KO*^ retinas showed an average of 4 crossings of major arteries and veins and many more crossings in smaller vessels, consistent with prior reports^18^, which was then strongly reduced by L6-F4-2 versus PBS or no injection (Supplementary Fig. 2a, b). *Ndp*^*KO*^ retinal vessels have been reported to exhibit increased filopodia^48^. At P8, *Ndp*^*KO*^ mice displayed elevated filopodia in *Ndp*^*KO*^ retinas and were rescued by L6-F4-2 treatment (Supplementary Fig. 2c, d). Single IVT injection of tetravalent WNT surrogates recognizing FZD_1/2/7_ or FZD_5/8_ at P0 did not rescue *Ndp*^*KO*^ retinal vein or artery phenotypes at P8, confirming the specific efficacy of the FZD_4_-selective L6-F4-2 agent (Supplementary Fig. 3).

### L6-F4-2 activates retinal endothelial WNT/β-catenin signaling

**Fig. 3:**
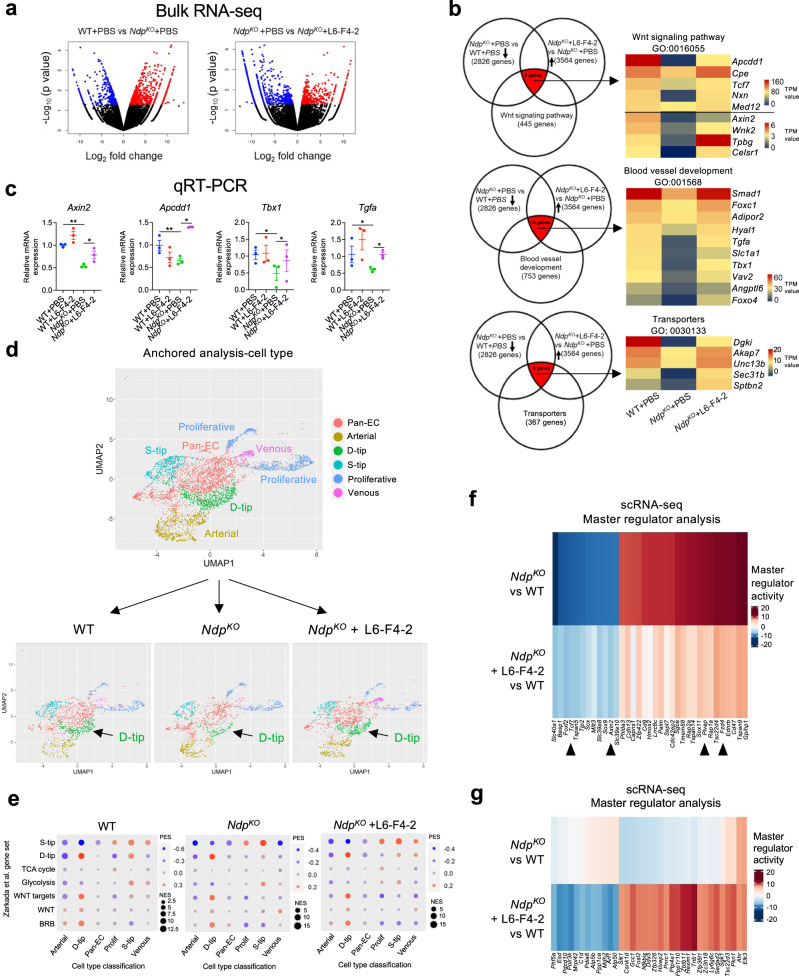
Effects of L6-F4-2 on retinal endothelial WNT/β-catenin signaling and vascular subtypes. **a**–**c** WT (control) and *Ndp*^*KO*^ male and female mice were treated with PBS or L6-F4-2 by intravitreal injection (0.19 μg) at P0 and 5-7 pooled retinas for each group were harvested at P8. Retinal vessel ECs were purified by anti-CD31 FACS and ultra-low input bulk RNA-seq analysis was performed. **a** Left: WT (PBS-treated) vs *Ndp*^*KO*^ (PBS-treated). Right: *Ndp*^*KO*^ (PBS-treated) vs *Ndp*^*KO*^ (treated with L6-F4-2). Volcano plot depicting genes with a p-value <0.05 and log_2_ fold-change > 1 are indicated by red dots which represent upregulated genes. Genes with a p-value <0.05 and log_2_ fold-change < −1 are indicated by blue dots, representing downregulated genes. **b** Left: Venn diagrams showing overlap in bulk RNA-seq differentially expressed genes following treatment with PBS or L6-F4-2 in WT or *Ndp*^*KO*^ mice. Right: Heat maps of the top expressed genes mapping to the indicated GO terms across treatment groups in the RNA-seq experiment. **c** qRT-PCR validation of differentially expressed genes identified by RNA-seq. Error bars represent mean ± SEM, triplicate from 5-7 pooled retina EC cells, *p < 0.05, **p < 0.01; one-way ANOVA. **d**–**f** Single-cell RNA-seq with the same treatment conditions as (a-c). **d** scRNA-seq UMAP plots of retinal EC sub-clusters from P8 retinas from all conditions merged (top) and from the WT, *Ndp*^*KO*^, and *Ndp*^*KO*^ + L6-F4-2 conditions (bottom), 5–7 pooled retinas for each group. Alteration of the D-tip population is indicated by arrows. **e** Dot plot of NaRnEA enrichment for previously described Zarkada et al. gene sets^50^. Dot size is determined by the absolute value of the normalized enrichment score (NES); large dots indicate more significant enrichment. Dot color is determined by the proportional enrichment score (PES), which indicates the sign of the enrichment. BRB, brain-retinal-barrier. **f** Master regulators of the *Ndp*^*KO*^ condition versus WT as identified by PISCES analysis of the scRNA-seq data with reduced or reverted activity profiles in the *Ndp*^*KO*^ + L6-F4-2 condition. **g** Master regulators of the *Ndp*^*KO*^ + L6-F4-2 condition versus WT without significant activity in the *Ndp*^*KO*^ condition as identified by PISCES analysis of scRNA seq data. Source data are provided as a Source Data file.

We next examined L6-F4-2 action by bulk RNA-seq of retinal endothelium from PBS or L6-F4-2-treated WT versus *Ndp*^*KO*^ mice, after single IVT injection at P0, retina harvest at P8 and FACS isolation of CD31 + ECs. This bulk RNA-seq identified retinal EC mRNAs corresponding to WNT target genes that were decreased by *Ndp*^*KO*^ mice but re-induced by L6-F4-2 (Fig. 3a, b), consistent with the diminution of WNT/β-catenin signaling activity in *Ndp*^*KO*^ mice^10^ and the rescue of *Ndp*^*KO*^ retinal angiogenesis by L6-F4-2 (Fig. 2). This *Ndp*^*KO*^- and L6-F4-2-regulated gene set overlapped with blood vessel development genes (GO:001568) and numerous established WNT/β-catenin signaling mRNAs (*Apcdd1, Axin2, Tcf7, Celsr1*; GO:0016055) including those known to be WNT-regulated in BBB ECs^15,49^ (Fig. 3b). We re-validated these findings by qRT-PCR of CD31 + retinal ECs, indicating that the expression of the WNT downstream target genes, *Axin2* and *Apcdd1*, and blood vessel development-related genes *Tbx1* and *Tgfa* were all reduced in *Ndp*^*KO*^ mice but rescued by L6-F4-2 (Fig. 3c).

### L6-F4-2 rescue of endothelial D-tip cells in *Ndp*^*KO*^ retina

We then performed single-cell RNA sequencing (scRNA-seq) on FACS-purified CD31+ P8 retinal ECs from *Ndp*^*KO*^ versus WT mice after P0 IVT injection of L6-F4-2 or PBS. These scRNA-seq data were analyzed for changes in endothelial subsets between the WT, *Ndp*^*KO*^, and *Ndp*^*KO*^ + L6-F4-2 treatment conditions. We first used the Seurat pipeline to anchor the retinal endothelial scRNA-seq data from the three experimental conditions, focusing on the >95% of sorted cells expressing the pan-endothelial markers *Cdh5 and Pecam1* to generate cell clusters in UMAP space. Prior single-cell RNA-seq has defined several neonatal retinal vascular cell types, including two classes of tip cells, which remain superficially (S-tip) on the retinal surface or dive (D-tip) into the neural retina respectively, as well as capillary, venous, arterial, and proliferative populations^50^. We thus applied the markers from this prior retinal endothelial classification^50^ to assign cell type identities to our unsupervised scRNA-seq Seurat analysis (Fig. 3d; Supplementary Fig. 4a, b). This identified *Cldn5*^*+*^*Mfsd2a*^*+*^*Spock2* + D-tip, *Angpt2*^*+*^*Esm1*^*+*^ S-tip, *Unc5b*^*+*^*Bmx*^*+*^ arterial, *Ptgis*^*high*^ venous and two clusters of *Mki67*^*+*^*Birc5*^*+*^ proliferative endothelium (Fig. 3d; Supplementary Fig. 4a, b). The remaining cells expressed *Cdh5 and Pecam1* but were not enriched for markers of other clusters or for previously described capillary markers^50^ and therefore were described as pan-endothelial (Pan-EC) (Fig. 3d, Supplementary Fig. 4a, b). Gene set variation analysis (GSVA) using NaRnEA^51^ against all three merged treatment conditions confirmed that D-tip and S-tip cells in our scRNA-seq analysis shared GSEA processes with previously described retinal D-tip and S-tip cells^50^, further supporting these cell type assignments. Besides this global overlap, the strongest associations for WNT signaling, glycolytic processes and TCA cycle were with D-tip, S-tip and proliferative subsets, respectively (Fig. 3e), consistent with prior reports^50^. The NaRnEA gene set enrichment did not differ between treatment conditions, suggesting that WNT signaling manipulation did not alter specific pathways within retinal cell types but rather changed the relative abundance of these populations. Our studies did not allow high-confidence identification of previously described capillary subsets^50^, which may reside in the pan-EC cluster (Fig. 3d, Supplementary Fig. 4a, b).

We focused on changes in cell type frequency between the identified populations in WT, *Ndp*^*KO*^ and *Ndp*^*KO*^ + L6-F4-2 using a chi-squared test, leveraging standardized residuals to identify significantly altered populations. By far, the most dominant effect between WT and *Ndp*^*KO*^ was significant depletion in D-tip cells (*p* = 7.4 ×10^−13^), which was subsequently rescued by L6-F4-2 (*p* = 1.2 × 10^−8^) (Fig. 3d). These changes in D-tip endothelium were consistent with (i) the strongly decreased vascularization of deep retinal layers in *Ndp*^*KO*^ and subsequent rescue by L6-F4-2 in vivo (Fig. 2e) and (ii) the finding that D-tip cells were also the subset having the strongest enrichment in WNT target genes (Fig. 3e). S-tip cells were less significantly decreased in *Ndp*^*KO*^ (*p* = 0.002) and not rescued by L6-F4-2 (p = 0.44) (Fig. 3d), potentially consistent with similar numbers of superficial endothelium, albeit with differential radial extension, in these three conditions (Fig. 2e).

### Master regulator analysis of L6-F4-2 action in retinal vasculature

We further stratified the scRNA-seq data into transcriptional nodes using master regulator analysis. A gene expression signature was generated for *Ndp*^*KO*^ v. WT and *Ndp*^*KO*^ + L6-F4-2 v. WT using the DESeq2 package in R. We then applied the PISCES (Protein Activity Inference in Single Cells) pipeline, to first generate a transcriptional network using ARACNe-AP^52^ (Algorithm for the Reconstruction of Accurate Cellular Networks with Adaptive Partitioning) from the WT data with *k* = 5 metacells, and then inferred protein activity using VIPER^53^ (Virtual Inference of Protein Activity by Enriched Regulon Analysis) to detect concomitant changes in downstream target mRNAs. The top such inferred proteins in each scRNA-seq experimental condition were selected as candidate master regulators (MRs), and these were compared to identify which significant retinal endothelial MRs in the *Ndp*^*KO*^ condition reverted to a more WT-like state upon L6-F4-2 rescue (*Ndp*^*KO*^ + L6-F4-2). QC metrics indicated robust library preparation (Supplementary Fig. 5), and the data were filtered based on thresholds in Supplementary Table 1.

The most downregulated MR activities in *Ndp*^*KO*^ mice (Fig. 3f, left column, deep blue) were substantially rescued by L6-F4-2 treatment of *Ndp*^*KO*^ mice (Fig. 3f, left column, light blue). Indeed, this unbiased analysis again revealed *Axin2* and *Tcf7* as MRs decreased in *Ndp*^*KO*^ but re-induced by L6-F4-2 (Fig. 3f), identical to bulk RNA-seq and qRT-PCR data (Fig. 3a–c). Plasmalemma vesicle-associated protein (PLVAP) is an EC fenestration component that regulates BBB/BRB permeability and is upregulated in pathological conditions associated with compromised barrier function and is decreased by WNT/β-catenin signaling^49,54,55^. Accordingly, *Plvap* was an inferred MR that was indeed overexpressed in *Ndp*^*KO*^ versus WT mice (Fig. 3f, right column, red), but downregulated by superimposed L6-F4-2 treatment (Fig. 3f, right column, pink). Interestingly, the MR *Fzd4* was increased in *Ndp*^*KO*^ but suppressed by L6-F4-2, potentially consistent with a feedback loop where absence of the Norrin ligand elicits compensatory upregulation of the receptor FZD_4_ and its signaling network, which is rescued by L6-F4-2 (Fig. 3f, right column). We also identified MRs which were substantially dysregulated instead of rescued by L6-F4-2 (Fig. 3g).

### L6-F4-2 treatment promotes BBB and BRB function in neonatal and young mice

Norrin/FZD_4_/WNT signaling is required for the development and maintenance of CNS endothelial cell barrier properties. Constitutive KO of *Ndp or* FZD_4_ elicits BBB permeability in cerebellum, which can be rescued by EC-specific transgenic expression of stabilized β-catenin or transgenic restoration of Norrin expression^11,18^. Further, FZD_4_ conditional KO in retinal ECs induces BRB leak^11,18^. We thus asked if L6-F4-2 could represent a therapeutic agent for CNS endothelial barrier dysfunction. Pharmacokinetic analysis of L6-F4-2 after i.p. or i.v. administration for bioavailability and clearance information suggested an extended in vivo half-life for L6-F4-2 of approximately 3 days, as expected of an antibody molecule (Supplementary Fig. 6).

**Fig. 4:**
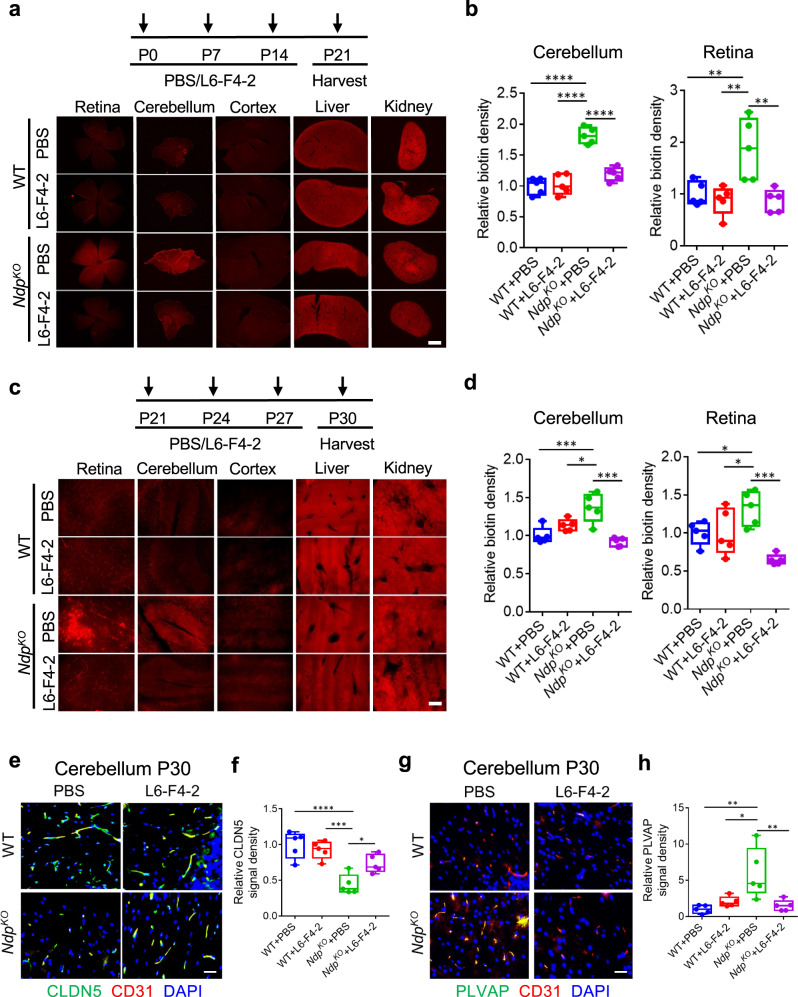
L6-F4-2 treatment promotes endothelial blood-brain and blood-retina barrier function. **a** WT (control) and *Ndp*^*KO*^ male and female mice were treated with PBS or L6-F4-2 by i.p. injection (2.5 mg/kg) at P0, P7 and P14 and Sulfo-NHS-biotin injected 30 min prior to tissue harvest at P21. Sulfo-NHS-biotin staining from a representative mouse reveals BBB/BRB defects in cerebellum and retina, but not cerebral cortex in *Ndp*^*KO*^ mice, while these defects can be rescued by L6-F4-2 treatment. Biotin labeling of liver and kidney parenchyma serve as a positive control. Scale bar, 500 μm. **b** Quantification of (**a**) for BBB/BRB leakage in *Ndp*^*KO*^ cerebellum and retina with rescue by L6-F4-2 treatment. **c** Mice were treated at P21, P24 and P27 (2.5 mg/kg, i.p.) and Sulfo-NHS-biotin injected 30 min prior to tissue harvest at P30. Sulfo-NHS-biotin staining from a representative mouse reveals BBB/BRB defects in cerebellum and retina but not cerebral cortex in *Ndp*^*KO*^ mice with rescue by L6-F4-2. Scale bar, 200 μm. **d** Quantification of (c) with BBB/BRB leakage in *Ndp*^*KO*^ cerebellum and retina and reversal by L6-F4-2. **e** L6-F4-2 rescues barrier phenotypes in P30 *Ndp*^*KO*^ mice with increased expression of the tight junction component CLDN5, P30 cerebellum IF, overlay of CD31 IF and DAPI. **f** Quantification of (**e**). **g** L6-F4-2 decreases expression of the EC fenestration component PLVAP in P30 *Ndp*^*KO*^ mice, with overlay of CD31 IF and DAPI. **h** Quantitation of (**g**). For (**e**) and (**g**), scale bars represent 100 μm. To quantify CLDN5 and PLVAP in (**f**) and (h) the density was measured with ImageJ and normalized to vessel area (CD31). Error bars in (**b**), (**d**), (**f**), and (**h**) represent mean ± SEM, *n* = 5 mice, *p < 0.05, **p < 0.01, ***p < 0.001, ****p < 0.0001; one-way ANOVA. Source data are provided as a Source Data file. For box plots, whiskers indicate the minimum and maximum values in the dataset. The center is the median number. Boundaries of boxes are the first quartile and third quartile.

We then performed i.p. injection of L6-F4-6 or PBS into *Ndp* WT and KO mice at P0, P7 and P14 with harvest at P21. At 30 min before harvest, the intravascular tracer Sulfo-NHS-biotin was injected i.p. Unsurprisingly, Sulfo-NHS-biotin did not extravasate from the retinal and cerebellar vasculatures of postnatal WT mice, consistent with an intact BRB and BBB. In contrast, *Ndp*^*KO*^ retina and cerebellum exhibited substantial extravascular biotin leak, consistent with published phenotypes^11,18^. However, L6-F4-2 treatment markedly improved BRB and BBB function, reducing biotin extravasation in *Ndp*^*KO*^ retina and cerebellum to essentially WT levels (Fig. 4a, b). *Ndp*^*KO*^ cerebral cortex did not exhibit biotin extravasation because of an intact BBB. Extensive vascular leak was observed in the parenchyma of WT and *Ndp*^*KO*^ liver and kidney, organs with fenestrated vasculature, which as expected was not significantly reversed by L6-F4-2 (Fig. 4a, b).

We studied if L6-F4-2 could restore *Ndp*^*KO*^ BBB and BRB defects in older mice by administering L6-F4-2 at P21, P24, P27 and harvesting tissues at P30. L6-F4-2 enhanced BRB and BBB barrier integrity in *Ndp*^*KO*^ mice, as quantification of biotin density in both cerebellum and retina revealed significant rescue by L6-F4-2 versus PBS (Fig. 4c, d). In WT mice, the cerebral cortex exhibited an intact BBB while fenestrated liver and kidney endothelium again manifested constitutive vascular leakage that was L6-F4-2-insensitive (Fig. 4c). The strong BBB defects in *Ndp*^*KO*^ mice were appropriately associated with reduced endothelial CLDN5 and increased PLVAP expression. However, L6-F4-2 treatment of *Ndp*^*KO*^ mice strongly upregulated CLDN 5 (Fig. 4e, f) and repressed PLVAP in endothelium of brain (Fig. 4g, h) and retina (Supplementary Fig. 7), indicating restoration of WNT/β-catenin signaling and barrier maturation. In total, these results indicated that L6-F4-2 not only reversed BRB phenotypes but also was robustly active in the BBB, indicating broad utility of FZD_4_ agonism.

### L6-F4-2 treatment rescues stroke phenotypes in adult WT mice

**Fig. 5:**
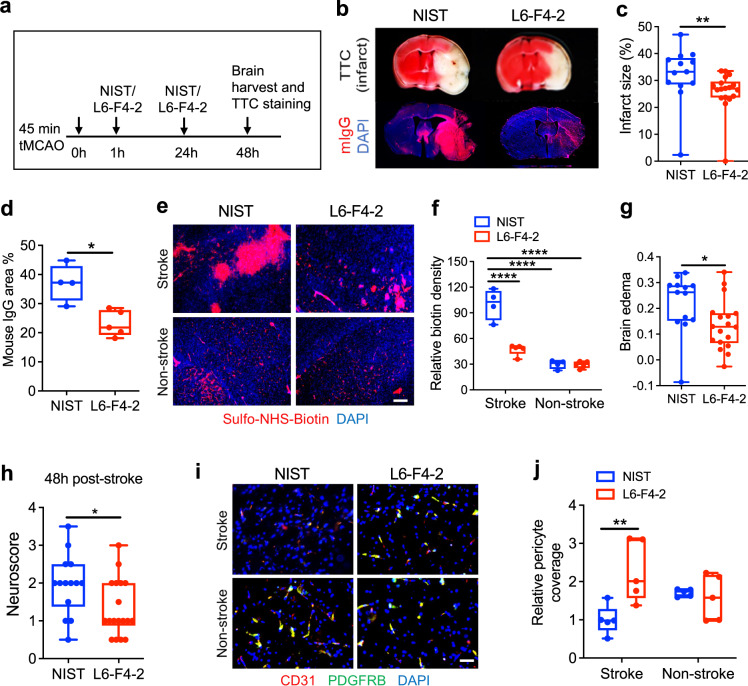
L6-F4-2 treatment rescues stroke and blood-brain barrier phenotypes in wild-type mice. **a** Schematic of tMCAO surgery with 45 min occlusion time, L6-F4-2 treatment time course and brain harvest at day 2 post-stroke. **b** Simultaneous TTC staining (top), and mouse IgG extravasation (mIgG) (bottom) analysis of coronal sections from representative mice at 48 h post-stoke. **c** Quantification of infarct size for NIST control mAb (*n* = 13 mice) and L6-F4-2 (*n* = 18 mice), males. *p* = 0.0042, two-sided Mann–Whitney *U* test. **d** Quantification of mouse IgG staining, NIST control *n* = 4 mice, L6-F4-2 *n* = 5 mice. *p* = 0.0159, two-sided Mann–Whitney *U* test. **e** Representative images of BBB leak in brains of the indicated mice in stroke and non-stroke regions after 45 min tMCAO, 2 days of reperfusion and two L6-F4-2 treatments (3 mg/kg, i.v.), as assessed by the Sulfo-NHS-biotin tracer extravasation assay. Scale bar, 50 μm. **f** Quantification of extravasated exogenous tracer Sulfo-NHS-biotin using ImageJ. *n* = 5 mice. **g** Fractional change in brain edema for NIST control mAb (*n* = 13 mice) and L6-F4-2 (*n* = 18 mice). *p* = 0.0307, two-sided Mann–Whitney *U* test. **h** Neurological scores at 48 h after tMCAO surgery for NIST control mAb (*n* = 13 mice) and L6-F4-2 (*n* = 18 mice). *p* = 0.0312, two-sided Mann–Whitney U test. **i** Co-immunofluorescence staining for PDGFRB and CD31 in infarcted brain (stroke and non-stroke) regions. **j** Quantification of pericyte coverage from (**i**). The PDGFRB signal was normalized to CD31; *n* = 5 mice. Scale bar, 100 μm. Error bars represent mean ± SEM, *p < 0.05, **p < 0.01, ****p < 0.0001; two-sided Mann–Whitney *U* test was used to compare groups in (**c**), (**d**), (**g**) and (**h**). Comparisons between multiple groups were made using one-way ANOVA test in (**f**) and (**j**). Source data are provided as a Source Data file. For box plots, whiskers indicate the minimum and maximum values in the dataset. The center is the median number. Boundaries of boxes are the first quartile and third quartile.

To expand the potential applications of FZD_4_-selective WNT surrogates beyond *Ndp*^*KO*^ mice to prevalent medical conditions, we investigated the effect of L6-F4-2 in adult ischemic stroke. Despite the substantial incidence of stroke-related death and disability^56^, treatment options are limited to mechanical stenting or pharmacologic therapy with tissue plasminogen activator (tPA), albeit with limited temporal windows and hemorrhage risk^57–59^. Wild-type adult C57Bl/6J mice (6–8 weeks) were subjected to transient middle cerebral artery occlusion stroke (tMCAO) by catheter occlusion to induce cerebral ischemia for 45 min, followed by reperfusion for 2 days. L6-F4-2 (3 mg/kg, i.v.) or control NIST mAb (National Institute of Standards and Technology, humanized IgG1κ monoclonal antibody) were administered at 1 and 24 h after tMCAO. Brains were harvested 48 h post-stroke and infarct TTC staining was immediately performed after tissue collection (Fig. 5a). The NIST and L6-F4-2 arms contained 20 mice each, of which *n* = 13 and *n* = 18 were evaluable after 48 h because of post-stroke mortality. L6-F4-2 substantially decreased cerebral infarct volume versus control mice (control *n* = 13, L6-F4-2 *n* = 18, *p* < 0.01) (Fig. 5b, c). Stroke-induced BBB permeability was assessed by IgG staining as a measure of plasma protein leak into the brain parenchyma. Accordingly, L6-F4-2 significantly reduced cerebral IgG extravasation versus NIST controls (control *n* = 13, L6-F4-2 *n* = 18, *p* < 0.05), consistent with BBB functional rescue (Fig. 5b, d). In addition, we assessed BBB integrity by Sulfo-NHS-biotin tracer extravasation. L6-F4-2 treatment also significantly alleviated the leakage of biotin in stroke regions, but no significant differences were observed in non-stroke areas (Fig. 5e, f). The L6-F4-2-induced rescue of infarct size and BBB integrity was further paralleled by significant reduction in edema (*p* < 0.05) (Fig. 5g) and neurological score (*p* = 0.0312) (Fig. 5h) versus NIST treatment.

Post-stroke effects of L6-F4-2 on neurovascular unit endothelial cells and pericytes, both of which are essential for BBB integrity, were characterized by IF staining of stroke and non-stroke hemispheres. Pericyte coverage of brain endothelium is markedly reduced post-stroke^15,60–62^ and the decreased pericyte coverage in the stroke hemisphere was strongly restored by L6-F4-2 treatment. In contrast, the contralateral non-stroke hemisphere in control and L6-F4-2-treated groups did not significantly differ in their substantial pericyte coverage of the endothelium (Fig. 5i, j).

## Discussion

The specialized blood-brain and blood-retinal barriers stringently regulate entry of substances from the circulation into the CNS parenchyma. The essential nature of these barrier functions is exposed by edema, hemorrhage, and inflammation during diverse CNS pathologies including neoplasia, neural degeneration, autoimmunity, and infection^3–6^. FZD_4_ is an attractive therapeutic target given its high expression in CNS endothelium^15,19,63^, essential functions in the BRB and cerebellar BBB, and functional redundancy with GPR124/RECK/WNT7 in cortical BBB regulation^10,11,18^. However, therapeutic use of FZD_4_ ligands is complicated by palmitoylation and insolubility of WNTs and difficulties in expressing Norrin^33,34^.

Here, we developed L6-F4-2, a selective tetravalent potent surrogate of the FZD_4_:LRP6 receptor complex. L6-F4-2 does not bind FZD_1-3_ or FZD_5-10_ ECDs but promotes the proximity of FZD_4_ to LRP6 coreceptors, consistent with our prior synthesis of bioengineered WNT surrogates targeting other FZD family members^35–38,41,64^. Compared to Norrin and the recently reported FZD_4_-selective WNT surrogate F4L5.13^28^, L6-F4-2 exhibits approximately 100 times higher FZD_4_ affinity and potency of WNT/β-catenin signaling activation in vitro. As opposed to the obligate palmitoylation of highly hydrophobic native WNTs, L6-F4-2 is non-lipidated, facilitating systemic delivery, efficacy, as well as ease of production. At the same time, FZD_4_ tropism may have further advantages of averting promiscuous activation of other FZD family members.

In vivo efficacy of L6-F4-2 is strongly supported by the robust rescue of retinal angiogenesis and BRB leakage in *Ndp*^*KO*^ newborn pups, where L6-F4-2 substitutes for the absent Norrin. In the *Ndp*^*KO*^ model, L6-F4-2-induced WNT-dependent gene expression in retinal endothelium, consistent with an on-target mechanism. Our scRNA-seq analysis revealed D-tip cells as the most significantly dysregulated endothelial cell type in *Ndp*^*KO*^ retina, which was indeed potently rescued by L6-F4-2 treatment. These findings are consonant with prior studies indicating particular enrichment of WNT signaling processes within D-tip cells^50^. scRNA-seq master regulator analysis revealed *Axin2* and *Tcf7* as MRs decreased in *Ndp*^*KO*^ but re-induced by L6-F4-2, identical to bulk RNA-seq and qRT-PCR data. PLVAP regulates BBB/BRB permeability and is upregulated in pathological conditions associated with compromised barrier function and is decreased by WNT/β-catenin signaling. Accordingly, *Plvap* was an inferred MR that was indeed overexpressed in *Ndp*^*KO*^ versus WT mice but downregulated by superimposed L6-F4-2 treatment. Interestingly, the MR *Fzd4* was increased in *Ndp*^*KO*^ but suppressed by L6-F4-2, potentially consistent with a feedback loop where the absence of the Norrin ligand elicits compensatory upregulation of the receptor FZD_4_ and its signaling network, which is rescued by L6-F4-2.

L6-F4-2 rescued numerous molecular features of the BRB, including Wnt signaling pathway, blood vessel development, and transporter genes. However, contrasting with the reversibility of barrier properties, we find it unlikely that L6-F4-2 would be able to rescue adult *Ndp*^*KO*^ phenotypes that originate in developmental defects, such as vision and capillary patterning. We utilized single intravitreal injection of L6-F4-2, analogous to intravitreal administration of VEGF inhibitors for macular degeneration and diabetic retinopathy^65^, in contrast to systemic treatment of retinal phenotypes with FZD_4_-selective WNT surrogates such as F4L5.13^28^. SZN-413, a FZD_4_-selective WNT surrogate distinct from L6-F4-2 with different antibody sequences and molecular formats and targets LRP5 instead of LRP6, has activity in mouse and rabbit BRB models^39^.

A GPR124-selective WNT7 mutant (K190A) selectively activates β-catenin signaling via GPR124/RECK without stimulating FZD, and also exhibits activity in experimental stroke models. However, WNT7 K109A is predicted to retain palmitoylation, which could strongly restrict circulating bioavailability and utility as a systemically administrative therapeutic. Indeed, adeno-associated virus (AAV) was used to directly express WNT7 K109A from virally-transduced neurons, endothelial cells and astrocytes, with the protein concentrating near brain capillaries^66^. Such viral vector-mediated local delivery of WNT7 K109A is distinct from the current studies where L6-F4-2 was administered into the mouse circulation, producing persistent pharmacokinetics and systemic BBB/BRB efficacy.

Lastly, our present studies extend the therapeutic utility of FZD_4_ agonism. Prior efforts have been restricted to studying WNT surrogates or transgenic Norrin expression in the retina^18,28,39^. Our results with L6-F4-2 extend the successful demonstration of FZD_4_ agonism from the BRB to additional indications in the blood-brain barrier, as evidenced by robust rescue of *Ndp*^*KO*^ cerebellar BBB permeability. L6-F4-2 also improved stroke outcomes and BBB function in wild-type adult C57Bl/6J mice, thus enlarging potential benefits beyond *Ndp/Tspan12* mutants or other WNT pathway-mutant hereditary retinopathies, to prevalent and disabling medical conditions in the general population such as cerebral infarction, as facilitated by our demonstration of successful intravitreal delivery. FZD_4_-selective WNT surrogates potentially could find broader general application to additional conditions such as neurodegeneration, neoplasia, autoimmunity, and infection, representing neurological diseases at-large characterized by BBB dysregulation. Further studies are needed to evaluate FZD_4_-selective WNT surrogate activity in these diseases.

## Methods

### Molecular cloning and protein generation

Constructs for F4-2_Fab (hereafter F4-2) and L6-F4-2 were cloned into the pcDNA3.1(+) mammalian expression vector (Thermo Fisher). The F4-2 heavy chain contains a 6xHis tag on its C-terminus. For the tetravalent constructs for L6-F4-2, each light chain variable region (VL) or heavy chain variable region (VH) of YW211.31.57 was fused onto the either light chain or heavy chain N-terminus of the F4-2 IgG through a 15-mer linker (GGGGSGGGGSGGGGS), which contains L234A/L235A/P329G mutations (LALAPG) to eliminate effector function^67^.

Recombinant proteins were produced in Expi293F cells (Thermo Fisher Scientific) by transient transfection. The F4-2_Fab protein was first purified using cOmplete® His-tag purification resin (Sigma-Aldrich), and L6-F4-2 protein was first purified with CaptivA Protein A affinity resin (Repligen). All proteins were further polished with Superdex 200 Increase 10/300 GL (GE Healthcare Life Sciences) size-exclusion chromatography (SEC) using 1 x HBS buffer (20 mM HEPES pH 7.4, 150 mM NaCl) or 2 x HBS buffer (40 mM HEPES pH 7.4, 300 mM NaCl). The CRDs of 10 FZDs were expressed and purified as previously described^41^. In brief, the CRDs of FZD1,2,4,5,7,8,10 were expressed in High Five cells (Invitrogen) and purified using Ni-NTA affinity purification. The CRDs of FZD3,6,9 were transiently produced in the Expi293 expression system (Thermo Fisher Scientific), purified by Protein A resin followed by HRV 3 C protease (Thermo Fisher) cleavage and SEC polishing. All proteins were also examined by SDS-polyacrylamide electrophoresis and estimated to be > 90% pure.

### Affinity measurement and binding specificity

Binding kinetics of F4-2_Fab or L6-F4-2 to FZD CRDs were determined by bio-layer interferometry (BLI) using an Octet Red 96 (PALL ForteBio, Fremont, CA) instrument at 30 °C, 1000 rpm with streptavidin (SA) biosensors. Biotinylated FZD_4_ CRD was diluted to 50 nM in the running buffer (PBS, 0.05% Tween-20, 0.5% BSA, pH 7.2) and captured to the SA biosensor followed by dipping into wells containing the F4-2_Fab or L6-F4-2 at different concentrations in running buffer or into a well with only running buffer as a reference channel. The dissociation of the interaction was followed with the running buffer. K_D_ for each binder was calculated by Octet System software, based on fitting to a 1:1 binding model. For binding specificity test, L6-F4-2 was diluted to 50 nM in the running buffer and captured to the AHC biosensor followed by dipping into wells containing 200 nM FZD CRDs in running buffer. The dissociation of the interaction was followed with the running buffer.

### Cell lines

HEK (ATCC, #CRL-3249), HRMECs (Cell systems, #ACBRl 181) and bEND.3 (ATCC, #CRL-2299) were maintained in DMEM (Sigma) supplemented with 10% fetal bovine serum (Sigma). All cells were cultured in humidified incubators at 37 °C and 5% CO2.

### SuperTop Flash (STF) assay

WNT/β-catenin signaling activity was measured using HEK293, HRMECs, and bEnd.3. In brief, WNT/β-catenin signaling activity was measured using HEK293 cells containing a luciferase gene controlled by a WNT-responsive promoter (Super Top Flash reporter assay [STF]), as previously reported^36^. The bEnd.3 cells were stably transfected with the STF plasmid and a constitutively expressed renilla luciferase. HRMECs were transiently transfected with the STF plasmid for the STF assay. Cells were seeded at a density of 10,000 per well in 96-well plates 24 h prior to treatment in the presence of 3 μM IWP2 to inhibit the production of endogenous WNTs. L6-F4-2 proteins were then added to the cells overnight. Recombinant human WNT3A (R&D Systems) and recombinant human Norrin (R&D Systems) were used as response comparators. Cells were lysed with Luciferase Cell Culture Lysis Reagent (Promega) and luciferase activity was measured with Luciferase Assay System (Promega) using vendor procedures.

### Purification and culture of mouse brain endothelial cells

Mouse brain endothelial cells were isolated from the cortex of adult C57BL/6 J mice by established procedures followed by in vitro puromycin selection and in vitro culture in EGM-2MV as described^15^. In brief, brains of 12–24-week-old adult mice were minced and disaggregated as described above. After centrifugation at 400 g for 5 min, cell pellets were resuspended in EGM-2 MV medium (cat. # CC3202, Clontech, Mountain View, CA) with 10% FBS and 4 μg/ml puromycin (to kill all other cell types except brain ECs) and plated into fibronectin-precoated plates (10 μg/ml for 30 min at 37 °C). 2–3 d later, medium was discarded, and cells were washed with PBS twice and medium was replaced with EGM-2 MV/10% FBS. The purity of isolated brain ECs was confirmed by CD31 and VE-cadherin immunofluorescence staining (>90% purity), with minimal contamination of pericytes (PDGFRβ-positive).

### Quantitative PCR analysis of gene expression

Either bEnd.3 cells or primary mouse brain endothelial cells were treated with 10 nM L6-F4-2 or NIST mAb for 24 h, followed by RNA extraction using the Qiagen RNeasy Micro Kit (Qiagen, Hilden, Germany) or the MagMAX mirVana Total RNA Isolation Kit (Thermo Fisher, A27828), respectively. cDNA was produced using the SuperScript IV VILO cDNA Synthesis Kit (Thermo Fisher, Waltham, MA). RNA was quantified using TaqMan Fast Advanced Master Mix with the following probes Mm00443610_m1 *Axin2*, Mm01278617_m1 *Mki67*, Mm01192208_m1 *Mfsd2a*, Mm00550265_m1 *Lef1* (Thermo Fisher, 4331182). Values were normalized to expression of constitutive *Actb* RNA using Mm02619580_g1 (Thermo Fisher, Waltham, MA).

RNA from FACS-sorted retinal endothelial cells was extracted using Arcturus PicoPure RNA Isolation Kit (Applied Biosystems) and reverse transcribed by the SuperScript IV VILO cDNA Synthesis Kit (Thermo Fisher, Waltham, MA). cDNA was pre-amplified using SsoAdvanced™ PreAmp Supermix (BioRad, Hercules, CA). qPCR was performed using SYBR green Master Mix with primers (5′ → 3′) as follows: *Axin2* (Forward: GCCGACCTCAAGTGCAAACTC, Reverse: GGCTGGTGCAAAGACATAGCC); *Apcdd1* (Forward: AACCCCACCTACACCCTCATC, Reverse: CGCCGTGAAGCTGGTAGTC); *Tbx1* (Forward: CTGTGGGACGAGTTCAATCAG, Reverse: TTGTCATCTACGGGCACAAAG); *Tgfa* (Forward: CACTCTGGGTACGTGGGTG, Reverse: CACAGGTGATAATGAGGACAGC) and *Cldn5* (Forward: GCGCCGGTCAAGGTAACAAAG, Reverse: ATGTCGTGCGTGGTGCAGAGT)^15^. Relative RNA expression was normalized to *Gapdh* (Forward: TGAACGGGAAGCTCACTGG, Reverse: TCCACCACCCTGTTGCTGTA).

### Mice

*Ndp*^*KO*^ mice (#012287) and WT mice (C57BL/6J #000664) were purchased from the Jackson Laboratory. Mice were housed and bred in a normal experimental room and exposed to a 12-hour light/dark cycle with free access to food and water. All procedures were performed in accordance with approved IACUC protocols at Stanford University (#12501) and Surrozen, Inc (#SRZ-IACUC-002).

### Intravitreal injection

For IVT injection in P0 male and female pups, animals were anesthetized via isoflurane induction. Once anesthetized and devoid of a pedal response, a small incision of ~1.5 mm was made with the tip of a surgical scalpel blade or straight vannas scissors along the eyelid margin. Curved forceps were used to poke the eye. The tip of a beveled needle was used to make a hole through the sclera into the vitreous space. A borosilicate glass tip was slid through the hole, and 0.5 µl L6-F4-2 (0.19 µg) or PBS was injected. The eye was gently pushed back into its socket, and a drop of lidocaine (2%) was administered.

### Whole-mount immunofluorescent staining and imaging of mouse retina

Mice were humanely sacrificed, and eyes were harvested and transferred into 4% paraformaldehyde (PFA) to fix for 10-15 min and then to cold PBS on ice in a Petri dish for dissection. Fat surrounding the eye was displaced. The edge of the cornea was pierced with sharp scissors and cut around the cornea and iris and discard. The lens and vitreous humor were removed using forceps. The hyaloid vessels were removed by gathering them in the center of the eye with fine forceps then quickly pulled away from the center of the retina. Then, 4 to 5 radial incisions reaching approximately 2/3 of the radius of the retina were created using spring scissors to create a ‘petal’ shape. PBS was drawn off using a Pasteur pipette to flatten the retina, and then excess PBS was removed with a small piece of absorbent paper. Cold (−20 °C) methanol was slowly dropped onto the retinal surface until covered and flooded with additional methanol, transferred into 48-well plates and gently rinsed in PBS. Next, PBS was removed, and retinas were covered with 100 μl of Perm/Block solution (PBS + 0.3% Triton + 0.2% BSA) for 1 h, incubated overnight at 4 °C with primary antibodies (Isolectin GS-IB4, Alexa Fluor™ 488 Conjugate or Anti-Actin, α-Smooth Muscle—Cy3™ antibody) and washed for 4 × 10 min in PBS + 0.3% Triton (PBSTX), all with gentle shaking. Retinas were then transferred onto slides using a wide bore plastic Pasteur pipette, excess PBSTX removed with absorbent tissue, and mounted using Prolong mounting media and set overnight. Retinas were imaged using a Zeiss LSM900 confocal microscope or a Keyence epifluorescence microscope.

### BBB perfusion assay, tissue processing, and immunohistochemistry

Male and female mice were injected intraperitoneally with Sulfo-NHS-biotin (200 µl of 20 mg/ml Sulfo-NHS-biotin in PBS) and sacrificed after 30 min. Tissues were harvested in 4% PFA in PBS overnight and hydrated in 30% sucrose in PBS at least for 24 h before embedding in OCT. Tissue sections of 100 μm thickness were cut using a vibratome. Tissues and retinas were stained with Cy3-Streptavidin and imaged using confocal or epifluorescence microscopes, quantified, and processed with ImageJ and Adobe Photoshop.

### Immunofluorescence analysis

For cerebellum histological analysis, tissues were processed the same as above in OCT. Tissue sections of 10 μM were blocked in 5% Normal Goat Serum (Jackson ImmunoResearch, West Grove, PA) in PBS + 1% BSA + 0.3% Triton X-100 for 1 h at room temperature. Samples were incubated at 4 °C with the following primary antibodies in PBS + 1% BSA + 0.3% Triton X-100 + 0.1% NaN_3_: hamster anti-CD31 (1:100, cat.#MAB1398Z, Millipore, Billerica, MA), rat anti-mouse PDGFRB (1:50, cat.#14-1402-82, Clone APB5, eBiosciences, San Diego, CA), rabbit anti-CLDN5 (1:100, cat.#34-1600, Thermo Fisher Scientific, MA), Rat anti-mouse PLVAP antibody, clone MECA-32 (Bio-Rad, Hercules, CA). Excess antibody was removed by rinsing 4x in PBS + 0.1% Triton X-100 for 10 min. Samples were then incubated at room temperature for 1 h with the following secondary fluorescently labeled antibodies: FITC or Cy3 goat anti-hamster IgG, FITC or Cy3 goat anti-rat IgG, FITC or Cy3 goat anti-rabbit IgG, Cy3 goat anti-mouse IgG, FITC or Cy3 streptavidin (Jackson ImmunoResearch, West Grove, PA) diluted 1:400 in PBS + 1% BSA + 0.3% Triton X-100 for 1 h at RT. Excess antibody was removed by rinsing 4× in PBS + 0.1% Triton X-100 for 10 min. Slides were mounted in Vectashield mounting medium with DAPI (Vector labs, Burlingame, CA) and imaged with an epifluorescence microscope to obtain 10×, 20× or 40× images. Immunofluorescence signal area or density was quantified by ImageJ and normalized by vessel area (CD31 signal area). Pericyte coverage was quantified by measuring the IF staining signal length of pericyte or endothelial profile with the NeuronJ plugin for ImageJ.

### Pharmacokinetics

The pharmacokinetic profiles of L6-F4-2 were assessed via intravenous (i.v.) and intraperitoneal (i.p.) routes of drug administration in adult mice (*n* = 3/group). Blood samples were obtained at 10 minutes, 6 h, 24 h, and days 2, 4, 7, and 14 after intravenous injection of L6-F4-2 at 3 mg/kg. Similarly, blood samples were obtained at 2 h, 6 h, 24 h, and days 2, 4, 7, and 14 after intraperitoneal injection of L6-F4-2 at 3 mg/kg. Serum was obtained from blood samples through centrifugation in MiniCollect® Tube 0.8 ml Z Serum Separator tubes (Greiner Bio-One). Serum samples were then assessed for L6-F4-2 using a human IgG ELISA Kit (ab195215, Abcam, MA), per manufacturer’s protocol. Pharmacokinetic parameters were estimated using noncompartmental analysis of serum L6-F4-2 concentrations in individual mice with sparse sampling using Phoenix WinNonlin. All animal experiments were performed according to national ethical guidelines in addition to the guidance and approval by the Institutional Animal Care and Use Committee (IACUC) of Surrozen, Inc. Euthanasia was conducted in compliance with the current requirements of The Guide for the Care and Use of Laboratory Animals, 8^th^ Edition, and AVMA Guidelines on Euthanasia. All animals were obtained from the Jackson Laboratory (Bar Harbor, ME). On arrival, animals were randomly assigned to group (5 animals/cage) housing and provided with rodent diet and water ad libitum. All mice were maintained on a 12:12-h light/dark photoperiod at an ambient temperature of 22  ±  2 °C.

### FACS sorting of retina vessel ECs

Retinas of P8 mice were harvested and pooled as described above. Fresh retinas were minced and incubated in 5 ml DMEM containing 200 U/ml collagenase I (Invitrogen) for 45 min at 37 °C with occasional shaking followed by filtering through a 40 µm nylon mesh. The cells were then centrifuged at 94 x g for 5 min at 4 °C and resuspended in PBS with 0.1% BSA + 2 mM EDTA. Endothelial cells were labeled with FITC rat anti-mouse CD31 (#553372, BD Pharmingen, NJ). 7-AAD (Invitrogen, Waltham, MA) were added to exclude dead cells. Staining was performed for 1 h at 4 °C. Retina ECs were sorted into DMEM medium with an Aria II sorter (BD) at the Stanford University Shared FACS Facility and FACS gating strategy is shown in Supplementary Fig. 8. Cells were further processed with single-cell RNA-seq Kit (10X Genomics, Pleasanton, CA) or with the Arcturus PicoPure RNA Isolation Kit (Applied Biosystems) for RNA extraction.

### Transient middle cerebral artery occlusion (tMCAO)

Adult WT C57Bl/6 J mice (males, 6-8 weeks old, JAX) were anesthetized with 2% isoflurane (30% oxygen/ 70% nitrous oxide), maintained at a surgical plane of anesthesia (1–1.5% isoflurane), and underwent middle cerebral artery occlusion by the intraluminal suture method for 45 min (body temperature maintained at 37 ± 0.5 °C)^68^. Animals had free access to food and water throughout the reperfusion period. Neurological score was evaluated at 48-h post-tMCAO by a blinded observer: 0, normal motor function; (1) flexion of torso and the contralateral forelimb upon lifting by the tail; (2) circling to the ipsilateral side but normal posture at rest; (3) leaning to the ipsilateral side at rest; (4) unable to walk spontaneously. After neurological testing, the mice were deeply anesthetized with isoflurane and the brains were sectioned into 2 mm-thick sections and placed in 2% 2,3,5 triphenyltetrazolium chloride (TTC, cat. #T8877, Sigma-Aldrich, St. Louis, MO) for 10 min at 37 °C to delineate infarcts. Infarct areas were determined using an image analysis system (ImageJ). Edema was calculated by the RICH technique by assessing fractional increase in volume of the stroke vs. ipsilateral non-stroke hemispheres^69^. The persons performing the surgery and recording neurological scores were blinded to treatment status.

### Bulk RNA sequencing

For retina ECs of WT and *Ndp*^*KO*^ mice with PBS or L6-F4-2 treatment, total RNA was extracted using the Arcturus PicoPure RNA Isolation Kit (Applied Biosystems). RNA-seq libraries were generated with the NEBNext Ultra II Directional RNA Library Prep Kit coupled with Poly(A) mRNA Magnetic Isolation Module and NEBNext multiplex oligos for Illumina (New England Biolabs). Deep sequencing was performed on the NextSeq 500 sequencing system (Illumina) with 75-cycle, paired-end sequencing. RNA data were aligned to Ensembl mouse genomes using Kallisto (v 0.44.0) with default parameters. Change in gene expression between two conditions was defined as significant if |log_2_FC| > 0.5 and adjusted *P* value <0.05. Complex-Heatmap was used to produce heat maps^70^.

### Single-cell RNA sequencing (scRNA-seq)

WT and *Ndp*^*KO*^ mice were treated with PBS or L6-F4-2 at P0. Retinas were harvested and retinal ECs were FACS sorted in DMEM without EDTA at P8. scRNA-seq with the 10X Genomics Single Cell 3′ platform was performed per manufacturer’s protocol. Cell capture, library preparation, and sequencing were performed as described^71^. In brief, the sorted cellular suspensions were loaded on a GemCode Single Cell Instrument (10x Genomics) to generate single-cell gel beads in emulsion (GEMs). Approximately 1,200–2,800 cells were loaded per channel. Single-cell RNA-seq libraries were prepared using GemCode Single Cell 3′ Gel Bead and Library Kit. Sequencing libraries were loaded at 2.1 pM on an Illumina Next-Seq500 with 2 × 75 paired-end kits using the following read length: 98 bp read1, 14 bp I7 index, 8 bp I5 index and 5 bp read2. Note that these libraries were generated before the official launch of GemCode Single Cell 3′ Gel Bead and Library Kit. Thus, 5 bp UMI was used (the official GemCode Single Cell 3′ Gel Bead contains 10 bp UMI).

### Transcriptional regulatory network analysis

A transcriptional regulatory network was reverse engineered using the WT data. Metacells were generated by summing the raw counts of each with those of its five nearest neighbors in SCT-normalized gene expression, using √1−*ρ* as the distance metric, where *ρ* is the Spearman correlation between samples. The data was then randomly subset to 500 such metacells, and the data was CPM normalized.

This CPM-normalized matrix of metacells was then used as input to ARACNe-AP^52^. Four regulator sets were used: TFs, coTFs, signaling, and surface proteins. ARACNe-AP was run using a p-value threshold of 10^−8^ with 200 bootstraps for each set of regulators, with the final network being consolidated from each of the four regulator-specific networks. The final network contained 3145 regulators with at least 50 edges that could be used for VIPER^53^ analysis.

The anchored data object consisting of joined data from the WT, *Ndp*^*KO*^, and *Ndp*^*KO*^ + L6-F4-2 samples was used as input to VIPER within the PISCES pipeline. The scaled data were extracted and used as a gene expression signature for input to VIPER along with the previously generated network. The final VIPER matrix consisted of protein activity inference for 955 proteins across the 4315 cells in the anchored dataset.

### Gene expression analysis

scRNA-seq data from the wild-type with PBS treatment, *Ndp*^*KO*^ with PBS treatment, and *Ndp*^*KO*^ with L6-F4-2 treatment conditions underwent quality control based on the minimum and maximum depth, minimum gene count, and maximum mitochondrial percentage values given in Supplementary Table 2. QC plots are displayed in Supplementary Fig. 5. Filtered counts were then loaded into Seurat objects following their standard pipeline. Data from the three experimental conditions were integrated with the Seurat anchoring pipeline. A UMAP reduction and clustering solution were generated with default parameters. Differential markers were identified with the ‘find_markers‘ function for each cluster.

### Gene Expression Visualization

We visualized the expression of retinal endothelial subset genes according to Zarkada et al. (ref. ^50^). First, we visualized the expression of the 13 genes used in ref. ^50^ to initially identify retinal endothelial subtypes. We then also visualized the top five differentially expressed markers for each cell type from ref. ^50^ within our scRNA-seq data. Because the previous described analysis^50^ was performed at both P6 and P10, we merged the differentially expressed genes from both of these time points (see Supplementary Tables S3A and S3B in ref. ^50^ for the entire gene sets) which were then compared to our data from P8 retina. Heatmaps were generated using the ‘complexHeatmap‘ package in R, and all other visualizations were generated using ‘ggplot2‘.

### NaRnEA analysis

To assist in mapping clusters to cell types, we utilized the same gene sets from Zarkada et al. (see Supplementary Table S4A in ref. ^50^) to perform enrichment analysis using the NaRnEA algorithm^51^. We parametrized our regulon by setting the association weight (AW) and association mode (AM) to one for all genes in the given sets. A signature was generated for each cluster via gene-level Wilcoxon Rank-Sum test for each cluster versus all others. Gene expression signature (GES) normalized enrichment scores (NES) were generated by transforming the p-value to a Z-score using ‘qnorm‘ and then assigning this Z-score based on the rank-biserial correlation. NaRnEA was then performed to identify enrichment of each gene set in the signature for each cluster.

### Statistical analysis of cell type frequency by experimental condition

A chi-square test was performed between the WT and *Ndp*^*KO*^ cell-type vectors as well as between the *Ndp*^*KO*^ and *Ndp*^*KO*^ + L6-F4-2 cell-type vectors using the ‘chisq.test‘ function in R. To extract cell-type level conclusions, we analyzed the standardized residuals for each group. P-values were corrected with the Benjamini-Hochberg procedure.

### Statistical analysis

Statistical analysis was performed using GraphPad Prism. All statistical tests used biological replicates and are indicated by group size (n) in figure legends. Results were expressed as mean ± SEM. (standard error of the mean). *p < 0.05, **p < 0.01, ***p < 0.001, ****p < 0.0001; Mann–Whitney *U* test was used to compare two groups and one-way ANOVA test was used to compare multiple groups.

### Reporting summary

Further information on research design is available in the Nature Portfolio Reporting Summary linked to this article.

## Supplementary information

Supplementary Information

Reporting Summary

## Data Availability

The bulk RNA-seq and scRNA-seq data sets generated in this study have been deposited in Gene Expression Omnibus with the accession code GSE223628 and GSE223498 individually. Bulk RNA data were aligned to Ensembl mouse genome using Kallisto (v 0.44.0) with default parameters. Source data and the raw data of graphs in the figures in this study is provided in Source Data files with this paper. Other raw data are available immediately upon request. Source data are provided with this paper.

## Source data

Source Data

## Code availability

Scripts to perform analyses of scRNA-seq data are provided with this paper. Custom code is available on GitHub (https://github.com/califano-lab/NDP-KO-SC) and on Zenodo as well http://zenodo.org/badge/latestdoi/592882572.
