# Supplementary material for: Therapeutic blood-brain barrier modulation and stroke treatment by a bioengineered FZD4-selective WNT surrogate in mice
Source: Nat Commun. 2023 Jun 2;14:2947. doi: 10.1038/s41467-023-37689-1 (PMC10238527; doi:10.1038/s41467-023-37689-1)
Supplement: Supplementary file 1 — Supplementary Information [file 41467_2023_37689_MOESM1_ESM.pdf]

Supplementary Information

Therapeutic blood-brain barrier modulation and stroke treatment by a bioengineered FZD_4-selective WNT surrogate in mice

Supplementary Figure 1

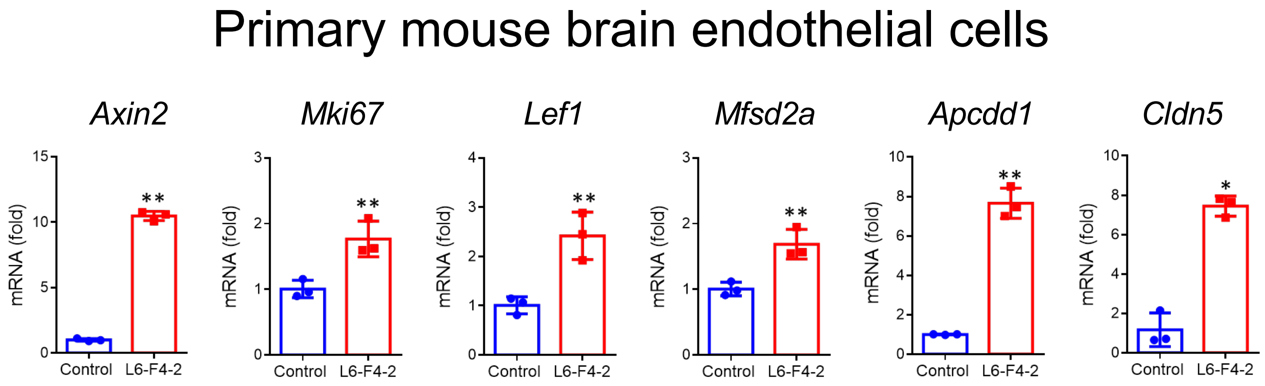

Supplementary Figure 1. L6-F4-2-stimulated gene expression in primary mouse brain endothelial cells. Primary brain endothelial cells were isolated from adult C57Bl/6J mice, cultured, and then treated with NIST (control) or L6-F4-2 (10 nM) for 24 hours. Cells were harvested, mRNA of indicated genes quantified by qRT-PCR, and expressed as fold-induction by L6-F4-2 relative to NIST. Error bars represent mean ± SEM, triplicate for pooled 2 mice, $^{*}p < 0.05$, $^{**}p<0.01$, two-sided Mann-Whitney U test. Source data are provided as a Source Data file.

Supplementary Figure 2

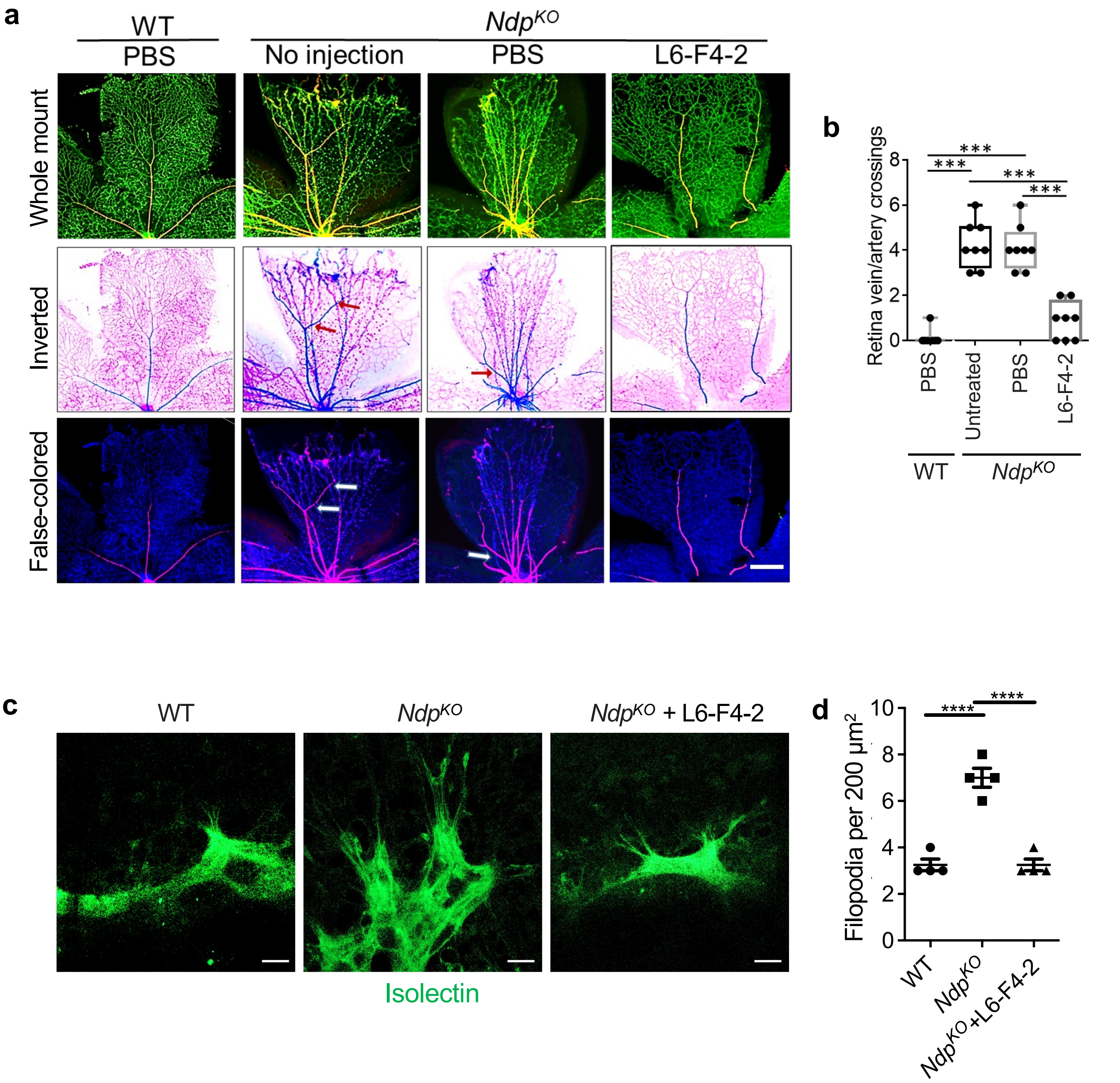

Supplementary Figure 2. Rescue of Ndp^{KO} retinal vascular phenotypes by L6-F4-2.
WT (control) and Ndp^{KO} mice were treated with PBS or L6-F4-2 by intravitreal injection (0.19 μg) at P0 and retinas were harvested at P8. (**a** and **b**) Isolectin B4-labeled veins (green/blue) and smooth muscle actin-labeled arteries (yellow/red) at P8 retina and quantification of crossings. Aberrant artery/vein crossings are seen in Ndp^{KO} and PBS treated Ndp^{KO} retinas (arrows), but not in the L6-F4-2-treated group. Error bars represent mean ± SEM, *Ndp* WT n=7 mice, Ndp^{KO} n=8 mice, ***p < 0.001; two-sided Mann-Whitney U test. **c** P8 retinas were stained with isolectin and filopodia quantified (**d**) in WT, Ndp^{KO} and Ndp^{KO}+L6-F4-2 mice. Scale bar, 200 μm. Error bars represent mean ± SEM, n=4 mice, ***p<0.001, ****p<0.0001, one-way ANOVA. Source data are provided as a Source Data file. For box plots, whiskers indicate the minimum and maximum values in the data set. The center is the median number. Boundaries of boxes are the first quartile and third quartile.

Supplementary Figure 3

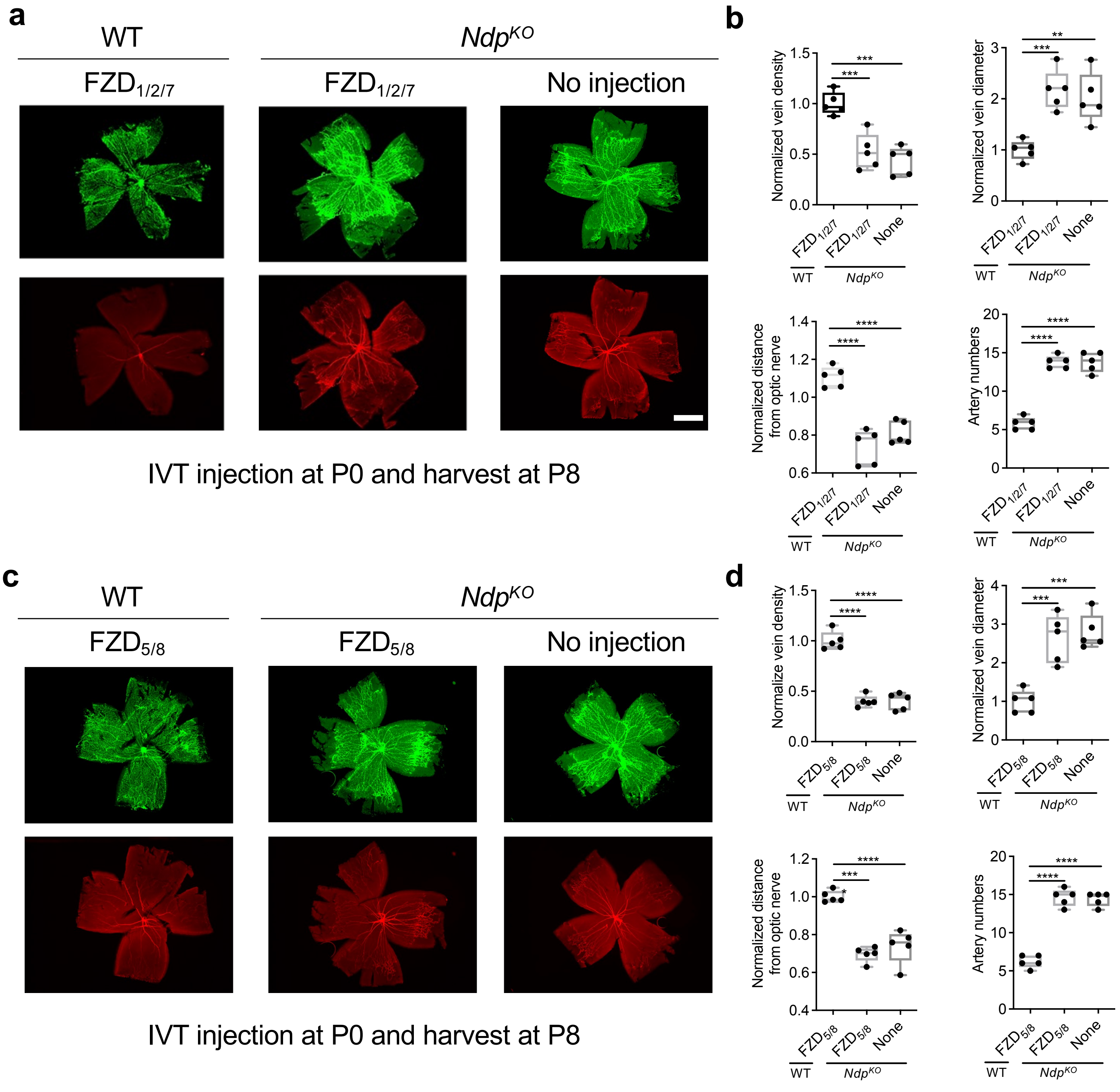

Supplementary Figure 3. $FZD_{1/2/7}$- and $FZD_{5/8}$-selective surrogates did not rescue retinal developmental angiogenesis in *Ndp*^{KO} mice. WT (control) and *Ndp*^{KO} mice were treated with PBS or FZD surrogates by intravitreal (IVT) injection (0.19 µg) at P0 with retina harvest at P8. $FZD_{1/2/7}$ surrogate (**a and b)** and $FZD_{5/8}$ surrogate (**c and d**) did not rescue abnormal retinal architectures. Isolectin B4-labeled veins (green) and anti-smooth muscle actin-labeled arteries (red) are depicted in retinal flat mounts. Scale bar = 0.5 mm. Error bars represent mean ± SEM, n=5 mice, **p<0.01, ***p<0.001, ****p<0.0001, one-way ANOVA. Source data are provided as a Source Data file. For box plots, whiskers indicate the minimum and maximum values in the data set. The center is the median number. Boundaries of boxes are the first quartile and third quartile.

Supplementary Figure 4

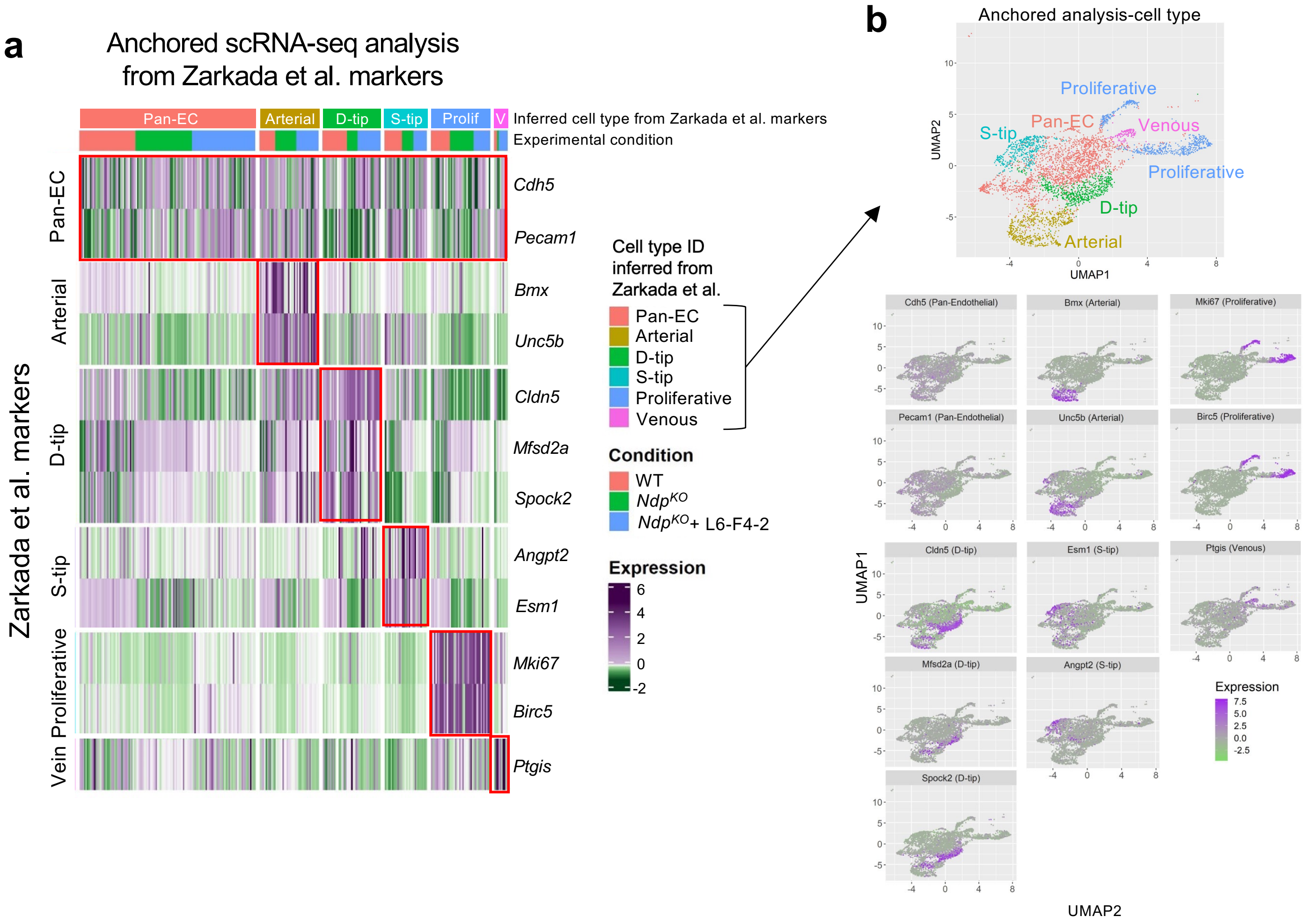

Supplementary Figure 4. a Heat map analysis of scRNA-seq data from Fig. 3d, displaying for each cluster SCT-normalized expression of cell type markers from Zarkada et al. (ref. [49]) in a Seurat-integrated dataset, containing all three of the WT, *Ndp*^{KO}, and *Ndp*^{KO} + L6-F4-2 experimental conditions. Concordance between Zarkada et al. markers and our scRNA-seq Seurat clusters identified *Cldn5*$^{+}$*Mfsd2a*$^{+}$*Spock2*$^{+}$ D-tip, *Angpt2*$^{+}$*Esm1*$^{+}$ S-tip, *Unc5b*$^{+}$*Bmx*$^{+}$ arterial, *Ptgis*^{high} venous and *Mki67*$^{+}$*Birc5*$^{+}$ proliferative endothelium in our scRNA-seq dataset. The remaining cells expressing *Cdh5* and *Pecam1* were described as pan-endothelial (pan-EC). **b** Feature plots labeling expression of the indicated genes within the pan-EC, D-tip, S-tip, arterial, proliferative, and venous populations identified from (**a**) in UMAP space, all WT, *Ndp*^{KO}, and *Ndp*^{KO} + L6-F4-2 experimental conditions merged. **c** Analysis of Fig. 3e with each treatment condition separated.

Supplementary Figure 5

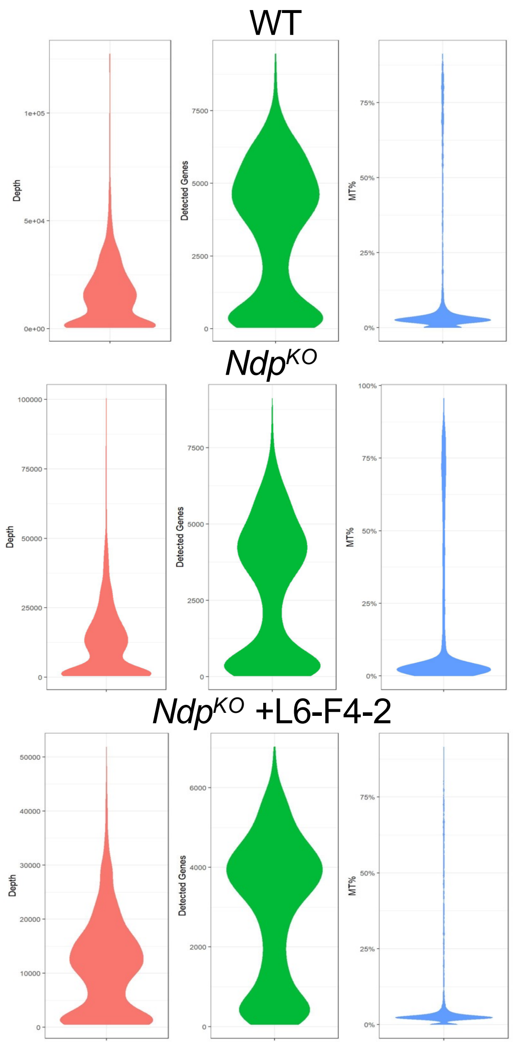

Supplementary Figure 5. Quality control metrics for scRNA-seq for each of the three experimental conditions. Values selected for filtration were selected based on this figure and can be found in Supplementary Table 1.

Supplementary Figure 6

a

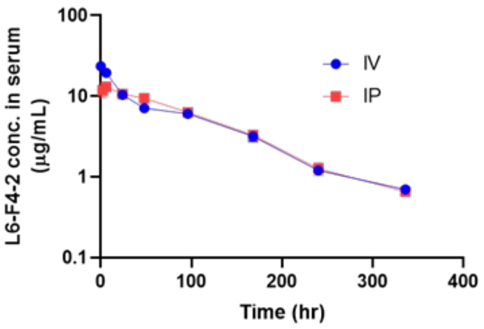

b

PK parameters

Dose Route	AUC_{inf} (ug-day/mL)	CL/F (mL/day/kg)	C_{max} (ug/mL)	V_{ss} (mL/kg)	Terminal half-life (days)
IP	64.2	46.7	13.0	211	3.10
IV	64.4	46.6	23.5	207	3.27

Supplementary Figure 6. Pharmacokinetics of L6-F4-2. a ELISA determination of the concentration–time profile of L6-F4-2 in wild-type mouse serum following a single 3 mg/kg intravenous (IV) or intraperitoneal (IP) dose (Mean ± SEM, n=3 mice per time point). **b** Mean PK parameters of L6-F4-2. F = bioavailability, AUC_{inf} = area under the curve to time infinity, CL = clearance, V_{ss} = volume of distribution at steady state, C_{max} = maximum observed concentration. The bioavailability after IP dosing was 100%.

Supplementary Figure 7

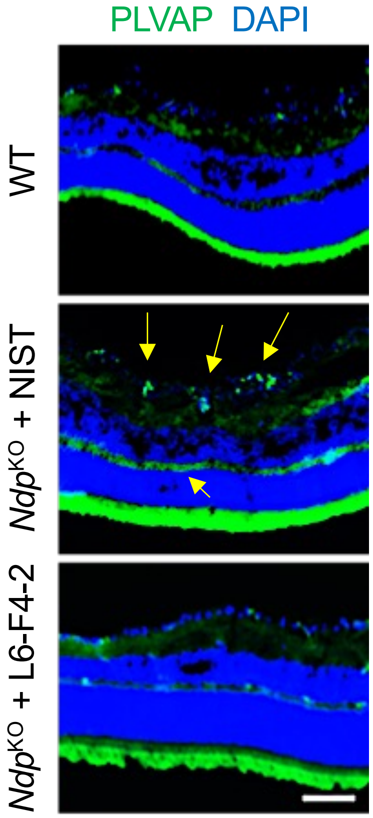

Supplementary Figure 7. L6-F4-2 administration decreased expression of PLVAP in *Ndp*^{KO} mice. *Ndp*^{KO} mice were treated with NIST or L6-F4-2 (2.5 mg/kg, i.p.) at P0, P7, P14 and retinas were harvested at P21. *Ndp*^{KO} mice inappropriately expressed PLVAP in the superficial and intermediate retinal layer (yellow arrows), which was rescued by L6-F4-2. Scale bar 100 μm.

Supplementary Figure 8

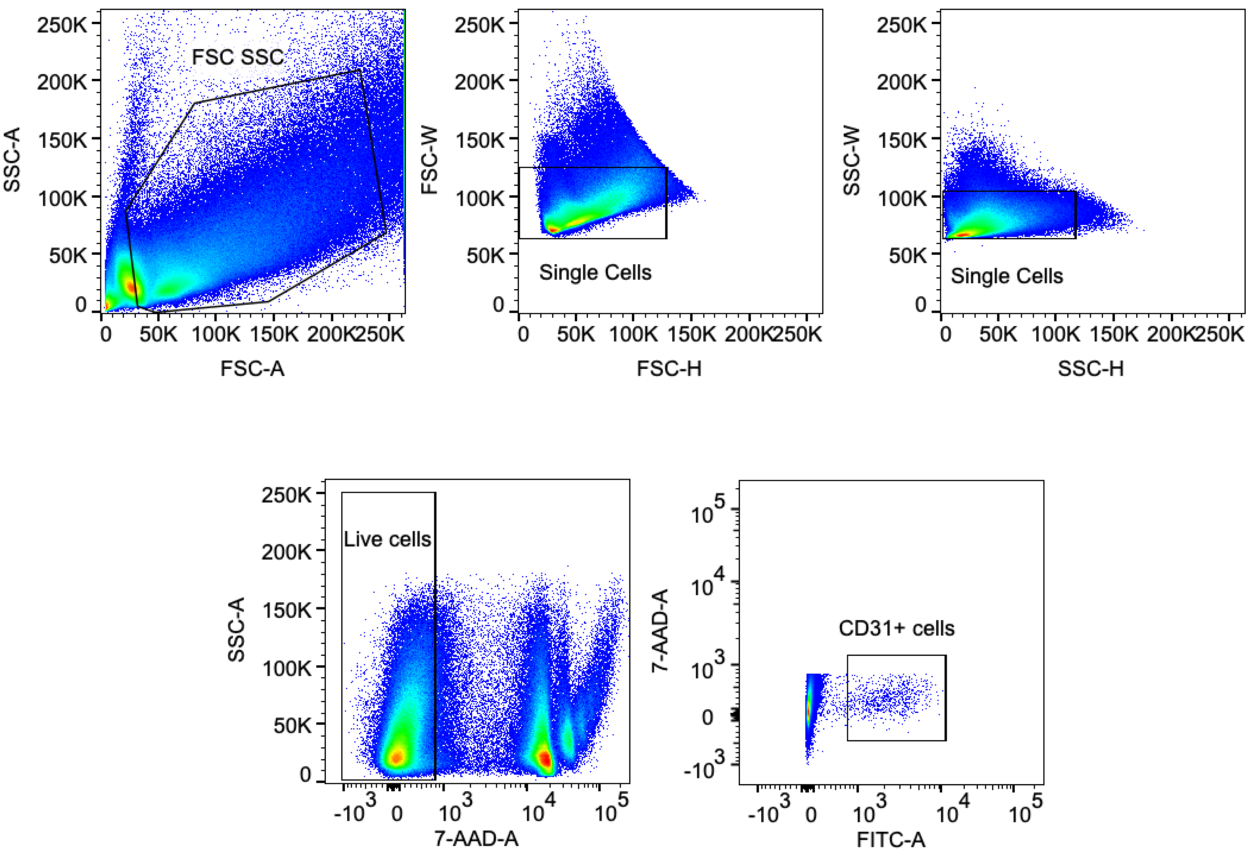

Supplementary Figure 8. FACS Cytometry gating strategy

All samples were initially gated using forward scatter (FSC) and side scatter (SSC) to identify events corresponding to cells, and then using FSC-W vs. FSC-H and SSC-W vs. SSC-H to enrich for single cells, next alive endothelial cells were selected by negativity for 7-AAD and positive for CD31.

Supplementary Table 1

	WT	*Ndp*^{KO}	*Ndp*^{KO} +L6-F4-2
**nCount_RNA**	>1250, <75000	>1000	>3000
**nFeature_RNA**	>1000	>1250	>1000
**Mt.features**	<12.5%	<25%	<12.5%

Supplementary Table 1. Filtration values used to subset scRNA-seq raw data in Seurat, based on QC metrics visualized in Supplementary Figure 5.

Supplementary Table 2

	min.depth	max.depth	min.genes	max.mat
**WT**	2000	50000	1000	0.1
***Ndp*^{KO}**	2000	50000	1000	0.1
Ndp*^{KO} + *L6-F4-2	2000	40000	1000	0.1

Supplementary Table 2. QC filtering values for each scRNA-seq experimental condition.
