# Supplementary material for: Therapeutic blood-brain barrier modulation and stroke treatment by a bioengineered FZD4-selective WNT surrogate in mice
Source: Nat Commun. 2023 Jun 2;14:2947. doi: 10.1038/s41467-023-37689-1 (PMC10238527; doi:10.1038/s41467-023-37689-1)
Supplement: Supplementary file 2 — Reporting Summary [file 41467_2023_37689_MOESM2_ESM.pdf]

nature portfolio

Corresponding author(s): Calvin Kuo

Last updated by author(s): Mar 2, 2023

Reporting Summary

Nature Portfolio wishes to improve the reproducibility of the work that we publish. This form provides structure for consistency and transparency in reporting. For further information on Nature Portfolio policies, see our Editorial Policies and the Editorial Policy Checklist.

Statistics

For all statistical analyses, confirm that the following items are present in the figure legend, table legend, main text, or Methods section.

n/a	Confirmed	
☐	☒	The exact sample size (*n*) for each experimental group/condition, given as a discrete number and unit of measurement
☐	☒	A statement on whether measurements were taken from distinct samples or whether the same sample was measured repeatedly
☐	☒	The statistical test(s) used AND whether they are one- or two-sided<br>*Only common tests should be described solely by name; describe more complex techniques in the Methods section.*
☒	☐	A description of all covariates tested
☒	☐	A description of any assumptions or corrections, such as tests of normality and adjustment for multiple comparisons
☐	☒	A full description of the statistical parameters including central tendency (e.g. means) or other basic estimates (e.g. regression coefficient) AND variation (e.g. standard deviation) or associated estimates of uncertainty (e.g. confidence intervals)
☐	☒	For null hypothesis testing, the test statistic (e.g. *F*, *t*, *r*) with confidence intervals, effect sizes, degrees of freedom and *P* value noted<br>*Give P values as exact values whenever suitable.*
☒	☐	For Bayesian analysis, information on the choice of priors and Markov chain Monte Carlo settings
☒	☐	For hierarchical and complex designs, identification of the appropriate level for tests and full reporting of outcomes
☒	☐	Estimates of effect sizes (e.g. Cohen's *d*, Pearson's *r*), indicating how they were calculated

Our web collection on statistics for biologists contains articles on many of the points above.

Software and code

Policy information about availability of computer code

Data collection	BD FACSDiva was used to collect postnatal day 8 retina endothelial cells.
Data analysis	We used GraphPad Prism 9 for data analysis. ImageJ 1.53a is used for microscopy images analysis . Bulk RNAseq data were analyzed by GENEWIZ and scRNA seq analysis codes are available on GitHub (https://github.com/califano-lab/NDP-KO-SC) and on Zenodo as well http://zenodo.org/badge/latestdoi/592882572. Bulk RNA data were aligned to Ensembl mouse genome using Kallisto (v 0.44.0) with default parameters.

For manuscripts utilizing custom algorithms or software that are central to the research but not yet described in published literature, software must be made available to editors and reviewers. We strongly encourage code deposition in a community repository (e.g. GitHub). See the Nature Portfolio guidelines for submitting code & software for further information.

Data

Policy information about availability of data

All manuscripts must include a data availability statement. This statement should provide the following information, where applicable:

- Accession codes, unique identifiers, or web links for publicly available datasets
- A description of any restrictions on data availability
- For clinical datasets or third party data, please ensure that the statement adheres to our policy

Data availability

The bulk RNA seq and scRNA-seq data sets generated in this study have been deposited in Gene Expression Omnibus with the accession code GSE223628 (https://www.ncbi.nlm.nih.gov/geo/query/acc.cgi?acc=GSE223628) and GSE223498 (https://www.ncbi.nlm.nih.gov/geo/query/acc.cgi?acc=GSE223498) individually. Source data All raw data of graphs in the figures in this study are provided in Source Data files with this paper. Bulk RNA data were aligned to Ensembl mouse genome using Kallisto (v 0.44.0) with default parameters.

Human research participants

Policy information about studies involving human research participants and Sex and Gender in Research.

Reporting on sex and gender	N/A
Population characteristics	N/A
Recruitment	N/A
Ethics oversight	N/A

Note that full information on the approval of the study protocol must also be provided in the manuscript.

Field-specific reporting

Please select the one below that is the best fit for your research. If you are not sure, read the appropriate sections before making your selection.

[x] Life sciences [ ] Behavioural & social sciences [ ] Ecological, evolutionary & environmental sciences

For a reference copy of the document with all sections, see nature.com/documents/nr-reporting-summary-flat.pdf

Life sciences study design

All studies must disclose on these points even when the disclosure is negative.

Sample size	No sample size calculation was performed. A minimum of 3 biological replicates was chosen for experiments for hypothesis testing against control groups to have at least two degrees of freedom.
Data exclusions	No data were excluded.
Replication	Replicate experiments were performed independently at least 3 times. Images or experiments showing technical replicates is representative of at least three experiments unless otherwise specified. Bulk RNA seq and single cell RNA-seq analysis was performed on pooled 5-7 mice per group. All replication attempts were successful.
Randomization	Mice were allocated into different groups based on the genotyping results. The allocations are random in other experiments.
Blinding	Neurological scores after tMCAO surgery were collected blindly. The animal experts who evaluated the neuro-scores were blind to the group allocation. In other experiments, the sample collections are not blind, but the data analysis is in a blinded manner.

Reporting for specific materials, systems and methods

We require information from authors about some types of materials, experimental systems and methods used in many studies. Here, indicate whether each material, system or method listed is relevant to your study. If you are not sure if a list item applies to your research, read the appropriate section before selecting a response.

Materials & experimental systems

n/a	Involved in the study	
[ ]	[x]	Antibodies
[ ]	[x]	Eukaryotic cell lines
[x]	[ ]	Palaeontology and archaeology
[ ]	[x]	Animals and other organisms
[x]	[ ]	Clinical data
[x]	[ ]	Dual use research of concern

Methods

n/a	Involved in the study	
[x]	[ ]	ChIP-seq
[ ]	[x]	Flow cytometry
[x]	[ ]	MRI-based neuroimaging

Antibodies

Antibodies used	All antibodies are commercially available and catalog numbers are provided in methods. Hamster anti-CD31 (1:100, cat.#MAB1398Z, Millipore, Billerica, MA), rat anti-mouse PDGFRB (1:50, cat.#14-1402-82, Clone APB5, eBiosciences, San Diego, CA), rabbit anti-CLDN5 (1:100, cat.#34-1600, Thermo Fisher Scientific, MA), Rat anti-mouse PLVAP antibody, clone MECA-32 (1:100, Bio-Rad, Hercules, CA). FITC or Cy3 goat anti-hamster IgG, FITC or Cy3 goat anti-rat IgG, FITC or Cy3 goat anti-rabbit IgG, Cy3 goat anti-mouse IgG, FITC or Cy3 streptavidin (Jackson ImmunoResearch, West Grove, PA). FITC rat anti-mouse CD31 (#553372, BD Pharmingen, NJ). 7-AAD (Invitrogen, Waltham, MA).
Validation	All primary antibodies were validated by the manufacturer in mice with additional citations in the primary literature. Hamster anti-CD31 (1:100, cat.#MAB1398Z, Millipore, Billerica, MA) was validated and cited in the manufacturer website (https://www.sigmaaldrich.com/US/en/product/mm/mab1398z). Rat anti-mouse PDGFRB (1:50, cat.#14-1402-82, Clone APB5, eBiosciences, San Diego, CA) was cited in the manufacturer website (https://www.thermofisher.com/antibody/product/CD140b-PDGFRB-Antibody-clone-APB5-Monoclonal/14-1402-82). Rabbit anti-CLDN5 (1:100, cat.#34-1600, Thermo Fisher Scientific, MA) was cited in the manufacturer website (https://www.thermofisher.com/antibody/product/Claudin-5-Antibody-Polyclonal/34-1600). Rat anti-mouse PLVAP antibody, clone MECA-32 (1:100, Bio-Rad, Hercules, CA) was cited in the manufacturer website (https://www.bio-rad antibodies.com/monoclonal/mouse-plvap-antibody-meca-32-mca2539.html?f=purified&JSESSIONID_STERLING=5CA1639C4CB96A0264A4C0079A24D836.ecommerce2&evCntryLang=US-en&cntry=US&thirdPartyCookieEnabled=true).

Eukaryotic cell lines

Policy information about cell lines and Sex and Gender in Research

Cell line source(s)	HEK293 (ATCC, #CRL-3249), HRMECs (Cell systems, #ACBRl 181) and bEND.3 (ATCC, #CRL-2299).
Authentication	Cell lines were thawed direcly from a cryopreserved aliquot purchased from ATCC/Cell systems.
Mycoplasma contamination	All cell lines were tested negative for mycoplasma.
Commonly misidentified lines (See ICLAC register)	N/A

Animals and other research organisms

Policy information about studies involving animals; ARRIVE guidelines recommended for reporting animal research, and Sex and Gender in Research

Laboratory animals	NdpKO mice (#012287) and WT mice (C57BL/6J #000664) were purchased from the Jackson Laboratory. For retina study, postnatal day 0-day 8 mice were used. For blood-brain barrier study, postnatal day 0-day 30 mice were used. For tMCAO study, 6-8 weeks mice were used. Mice were housed and bred in a normal experimental room and exposed to a 12-hour light/dark cycle with free access to food and water.
Wild animals	This study did not involve wild animals.
Reporting on sex	Male and female were equally used in all experiments except for tMCAO, where only male mice were tested.
Field-collected samples	This study did not involve field-collected samples.
Ethics oversight	All procedures were performed in accordance with approved IACUC protocols at Stanford University and Surrozen, Inc.

Note that full information on the approval of the study protocol must also be provided in the manuscript.

Flow Cytometry

Plots

Confirm that:

- [x] The axis labels state the marker and fluorochrome used (e.g. CD4-FITC).
- [x] The axis scales are clearly visible. Include numbers along axes only for bottom left plot of group (a 'group' is an analysis of identical markers).
- [x] All plots are contour plots with outliers or pseudocolor plots.
- [x] A numerical value for number of cells or percentage (with statistics) is provided.

Methodology

Sample preparation	Retinas of P8 mice were harvested and pooled as described above. Fresh retinas were minced and incubated in 5 ml DMEM containing 200 U/ml collagenase I (Invitrogen) for 45 mins at 37°C with occasional shaking followed by filtering through a 40

	µm nylon mesh. The cells were then centrifuged at 94 x g for 5 mins at 4°C and resuspended in PBS with 0.1% BSA + 2 mM EDTA. Endothelial cells were labeled with FITC rat anti-mouse CD31 (#553372, BD Pharmingen, NJ). 7-AAD (Invitrogen, Waltham, MA) was added to exclude dead cells. Staining was performed for 1 h at 4°C.
Instrument	Aria II sorter (BD)
Software	BD FACS Diva
Cell population abundance	Endothelial cells were labeled with FITC rat anti-mouse CD31 (#553372, BD Pharmingen, NJ). 7-AAD (Invitrogen, Waltham, MA) was added to exclude dead cells.
Gating strategy	All samples were initially gated using forward scatter (FSC) and side scatter (SSC) to identify events corresponding to cells, and then using FSC-W vs. FSC-H and SSC-W vs. SSC-H to enrich for single cells, next alive endothelial cells were selected by negativity for 7-AAD and positive for CD31.

☒ Tick this box to confirm that a figure exemplifying the gating strategy is provided in the Supplementary Information.
